# Systems biology identifies preserved integrity but impaired metabolism of mitochondria due to a glycolytic defect in Alzheimer's disease neurons

**DOI:** 10.1111/acel.12924

**Published:** 2019-02-21

**Authors:** Pierre Theurey, Niamh M. C. Connolly, Ilaria Fortunati, Emy Basso, Susette Lauwen, Camilla Ferrante, Catarina Moreira Pinho, Alvin Joselin, Anna Gioran, Daniele Bano, David S. Park, Maria Ankarcrona, Paola Pizzo, Jochen H. M. Prehn

**Affiliations:** ^1^ Department of Biomedical Sciences University of Padua Padua Italy; ^2^ Department of Physiology & Medical Physics Royal College of Surgeons in Ireland Dublin Ireland; ^3^ Department of Chemical Sciences University of Padua Padua Italy; ^4^ Neuroscience Institute – Italian National Research Council (CNR) Padua Italy; ^5^ Center for Alzheimer Research, Division of Neurogeriatrics, Department of Neurobiology, Care Sciences and Society Karolinska Institutet Stockholm Sweden; ^6^ Brain & Mind Research Institute University of Ottawa Ottawa Ontario Canada; ^7^ German Center for Neurodegenerative Diseases (DZNE) Bonn Germany

**Keywords:** Alzheimer's disease, glycolysis, mitochondria, neurons, systems biology

## Abstract

Mitochondrial dysfunction is implicated in most neurodegenerative diseases, including Alzheimer's disease (AD). We here combined experimental and computational approaches to investigate mitochondrial health and bioenergetic function in neurons from a double transgenic animal model of AD (PS2APP/B6.152H). Experiments in primary cortical neurons demonstrated that AD neurons had reduced mitochondrial respiratory capacity. Interestingly, the computational model predicted that this mitochondrial bioenergetic phenotype could not be explained by any defect in the mitochondrial respiratory chain (RC), but could be closely resembled by a simulated impairment in the mitochondrial NADH flux. Further computational analysis predicted that such an impairment would reduce levels of mitochondrial NADH, both in the resting state and following pharmacological manipulation of the RC. To validate these predictions, we utilized fluorescence lifetime imaging microscopy (FLIM) and autofluorescence imaging and confirmed that transgenic AD neurons had reduced mitochondrial NAD(P)H levels at rest, and impaired power of mitochondrial NAD(P)H production. Of note, FLIM measurements also highlighted reduced cytosolic NAD(P)H in these cells, and extracellular acidification experiments showed an impaired glycolytic flux. The impaired glycolytic flux was identified to be responsible for the observed mitochondrial hypometabolism, since bypassing glycolysis with pyruvate restored mitochondrial health. This study highlights the benefits of a systems biology approach when investigating complex, nonintuitive molecular processes such as mitochondrial bioenergetics, and indicates that primary cortical neurons from a transgenic AD model have reduced glycolytic flux, leading to reduced cytosolic and mitochondrial NAD(P)H and reduced mitochondrial respiratory capacity.

## 1 INTRODUCTION

Alzheimer's disease (AD) is the most common cause of dementia, accounting for up to 75% of all cases (Mayeux & Stern, 2012; Qiu, Kivipelto, & Strauss, 2009). Mutations in three genes have been identified in familial AD, encoding: amyloid precursor protein (APP), presenilin‐1 (PS‐1) and PS2 (Reitz & Mayeux, 2014). Interestingly, these proteins are involved in the production of amyloid β‐peptides (Aβ). This observation, and the description of Aβ toxicity, has led to the amyloid hypothesis for AD, which postulates that Aβ accumulation drives a cascade of events leading to progressive synaptic and neuronal dysfunction and, eventually, cell death (Hardy & Selkoe, 2002). This hypothesis has been strongly challenged, however.

In particular, the role of mitochondrial dysfunction in the aetiology of AD is of increasing interest (Ankarcrona, Mangialasche, & Winblad, 2010). Indeed, mitochondrial alterations have been described for many years in AD (Baloyannis, 2006; Bubber, Haroutunian, Fisch, Blass, & Gibson, 2005; Hirai et al., 2001). In particular, defects in complex IV activity have been frequently reported (Cardoso, Santana, Swerdlow, & Oliveira, 2004; Cottrell, Borthwick, Johnson, Ince, & Turnbull, 2002; Kish et al., 1992; Parker & Parks, 1990; Parker, Filley, & Parks, 1995). Nevertheless, the majority of these studies were performed in postmortem tissue from AD patients, or in a context where the clinical pathology was already apparent, thus not distinguishing between causal and consequential mitochondrial defects in AD.

Computational models are mathematical descriptions of the current state of knowledge, and have emerged as valuable tools in biology to enhance traditional hypothesis‐based experimental approaches (Brodland, 2015)*.* In order to provide a holistic molecular interpretation of experimental data and further inform experimental design, we integrated a multilevel assessment of mitochondrial function (Connolly et al., 2017) in a cellular model of AD in the absence of overt Aβ toxicity (Ozmen, Albientz, Czech, & Jacobsen, 2009), with thorough analysis of a flux‐based computational model of the mitochondrial respiratory chain (RC) (Beard, 2005; Huber, Dussmann, Kilbride, Rehm, & Prehn, 2011).

## 2 RESULTS

### 2.1 Calibration of a flux‐based computational model of the mitochondrial respiratory chain

**Figure 1:**
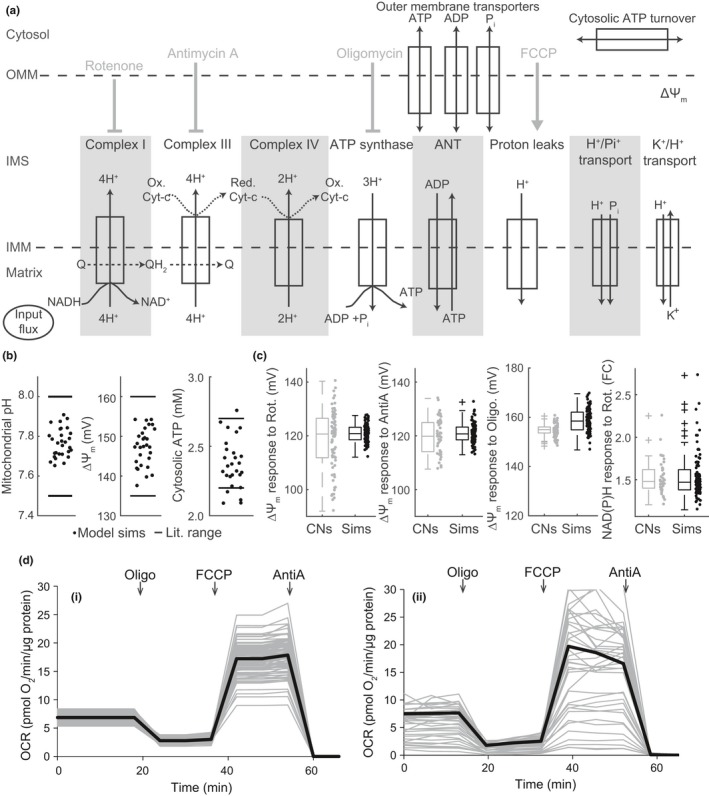
Parameterization and calibration of ordinary differential equation flux‐based model to experiments in primary cortical neurons from wild‐type (WT) mice. (a) Schematic indicating model compartments, modules and fluxes. Drug additions were simulated by altering the fluxes through the indicated modules. IMM, inner mitochondrial membrane; OMM, outer mitochondrial membrane; IMS, intermembrane space. (b) Simulated values (30 simulations, black dots) for mitochondrial pH, mitochondrial membrane potential (Δψ_m_) and cytosolic ATP concentration, compared to the range of values reported in the literature (black lines). (c) The simulated response (Sims; mV or fold change (FC) over baseline) of the mitochondrial membrane potential (ΔΨ_m_) to oligomycin (Oligo), rotenone (Rot) and antimycin A (AntiA) closely resembled TMRM and NAD(P)H autofluorescence measurements in WT primary cortical neurons (CNs; values compared 20 min after drug addition). Rotenone/antimycin A were simulated by reducing complex I/III activity respectively to 20% of unperturbed condition, oligomycin by reducing F_1_F_o_ ATP synthase activity to 13%, and FCCP by increasing H+ leak flux activity to 11*baseline flux. (d) The simulated flux through complex IV (Di), used as a proxy for the mitochondrial oxygen consumption rate, closely resembled oxygen consumption rate measurements in populations of WT primary cortical neurons (Dii) exposed to Oligo (2 μg/ml), FCCP (0.5 μM) and AntiA (1 μM). Traces represent individual simulations or wells. The mean of all traces is shown in black. Nonmitochondrial respiration has been subtracted from the experimental traces

We implemented a previously published (Beard, 2005; Huber et al., 2011) computational model of the mitochondrial RC that incorporates fluxes through the mitochondrial respiratory complexes, ATP production mediated by the F_1_F_o_ ATP synthase, the mitochondrial membrane potential, and nucleotide, ion and proton fluxes across the mitochondrial membranes (Figure 1a). The model is described in detail in Methods and Supporting Information Appendix S1. We first parameterized the computational model using values from the literature (preferentially from wild‐type (WT) primary neurons; see Supporting Information Tables S1–S4 for model description and literature references). Cell population simulations demonstrated that state variables in the basal (unstimulated) condition lay within the range of values reported in the literature (Figure 1b). We then simulated the addition of pharmacological agents by reducing the flux through the relevant respiratory complex (rotenone—complex I, antimycin A—complex III, oligomycin—F_1_F_o_ ATP synthase) or increasing the H^+^ leak across the mitochondrial inner membrane (simulating FCCP; Figure 1a). We next calibrated parameters to in‐house measurements of mitochondrial membrane potential (Figure 1c), mitochondrial NAD(P)H (Figure 1c) and oxygen consumption rate (Figure 1d) in WT mouse cortical neurons, and demonstrated that the computational model closely resembled the steady‐state responses of neurons exposed to various pharmacological inhibitors of the RC.

### 2.2 Transgenic AD neurons have impaired mitochondrial respiratory capacity

**Figure 2:**
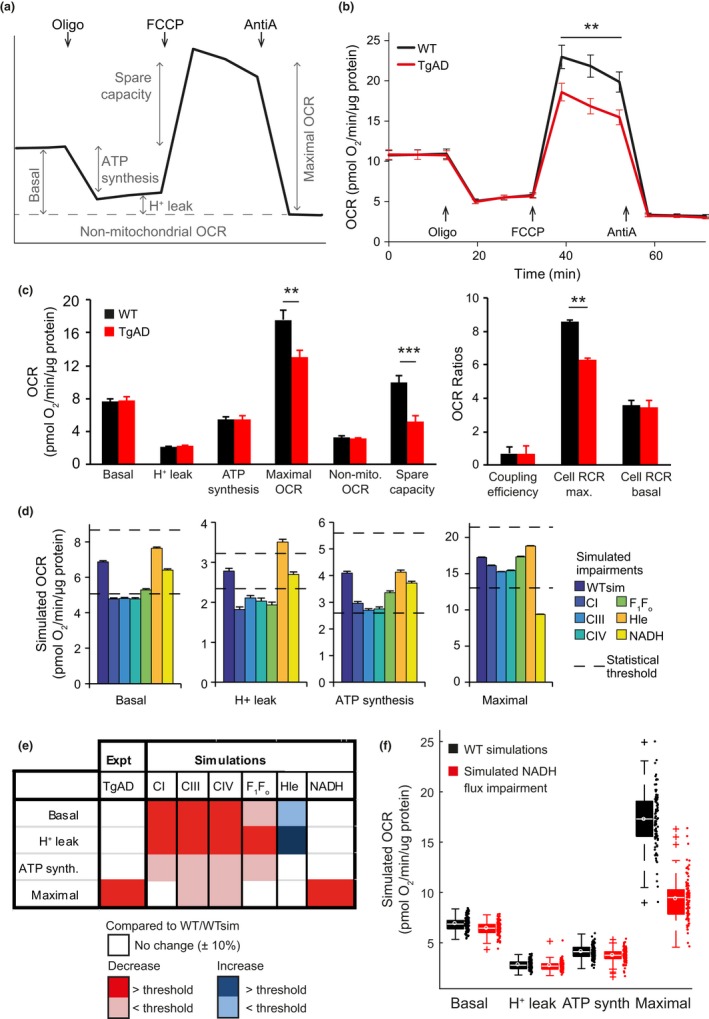
Oxygraphy measurements in live cells show impairment of maximal respiration in transgenic AD neurons, and the computational model predicts that this can be explained by a defect in mitochondrial NADH flux. (a) The experimental protocol followed to measure the oxygen consumption rate (OCR) in primary cortical neurons. The classical “mitochondrial stress test” assesses mitochondrial respiratory activity: oligomycin (Oligo, 2 μg/ml) inhibits the F_1_F_o_ ATP synthase, FCCP (0.5 μM) uncouples respiration, and antimycin A (AntiA; 1 μM) inhibits electron flow and abolishes mitochondrial respiration. Nonmitochondrial OCR (OCR remaining after addition of all drugs) is subtracted from all measurements to calculate the displayed OCR metrics (Brand & Nicholls, 2011): basal = mitochondria‐specific respiration at rest; ATP synthesis = basal OCR dedicated to ATP production (oligomycin‐sensitive respiration); proton (H^+^) leak = basal OCR uncoupled from ATP production (oligomycin‐insensitive respiration); maximal OCR = OCR upon uncoupling of respiration from ATP production; spare capacity = “spare” OCR available while at rest (maximal–basal). (b) Mean OCR measured in primary cortical neurons from wild‐type (WT) and transgenic AD (TgAD) mice. *p = 0.006. *n* (independent cultures‐number of wells): WT 7‐37, TgAD 8‐43. (c) OCR metrics calculated from measurements in WT and TgAD cortical neurons, as in (a). Coupling efficiency (= ATP synthesis/basal) reports the relative fluxes through the ATP synthase and proton leak pathways. Cell RCR max. and cell RCR basal (= maximal/H^+^ leak and basal/H^+^ leak, respectively) report the efficiency of substrate oxidation in the basal or maximal respiration state. **p < 0.01; ***p < 0.001. n: WT 10‐67, TgAD 12‐75. (d) Mean OCR metrics predicted by the computational model with no simulated impairment (WTsim), and impairments simulated in the indicated fluxes (100 simulations for each). Impairments were simulated by reducing fluxes to 70% of WTsim for respiratory complex I (CI), CIII, CIV and F_1_F_o_ ATP synthase; increasing the H^+^ leak flux (Hle) to 150% of WTsim; or reducing the NADH flux to 95% WTsim. The dashed lines indicate the statistical thresholds defined to identify whether predicted changes were likely to be measured experimentally (see Section 4). H+ leak and ATP synthesis are reported as oligomycin‐insensitive and oligomycin‐sensitive respiration, respectively, to allow direct comparison with experiments (see Supporting Information Appendix S1). (e) Heatmap highlighting the OCR metrics that differ between WT and TgAD neurons. The first column illustrates that only maximal respiration differed between WT and TgAD neurons in experimental measurements (shaded dark red). The subsequent columns indicate the changes predicted by the computational model with impairments simulated as in (d). Changes > ±10% compared to WTsim are marked light blue/red, while changes that exceed the statistically defined thresholds are marked dark blue/red. The model predicted that only an impairment in NADH flux could correctly reproduce the experimentally observed behaviour. (f) Predicted OCR metrics in WTsim compared to simulations with impaired NADH flux. The mean value is plotted as an unfilled circle within the boxplots (100 simulations)

Using a Seahorse XF Analyzer, we measured the oxygen consumption rate (OCR) in primary cortical neurons from both WT and B6.152H transgenic mice, a genetic model of AD (hereafter named transgenic AD [TgAD] mice). We performed the classical “mitochondrial stress test” protocol (Connolly et al., 2017; Figure 2a). Interestingly, basal OCR, the OCR contributing to ATP synthesis (oligomycin‐sensitive respiration) and the OCR consumed by H^+^ leak (oligomycin‐insensitive respiration) were similar in both WT and TgAD neurons (Figure 2b,c). Maximum OCR induced by mitochondrial uncoupling (FCCP), however, was significantly lower in TgAD neurons (−24%, *p* = 0.003; Figure 2c). Calculating additional bioenergetic metrics (Brand & Nicholls, 2011), we observed that TgAD neurons also exhibited reduced spare capacity and cell respiratory control ratio (−47%, *p* = 1 × 10^−5^ and −27%, *p* = 0.002, respectively; Figure 2c).

### 2.3 Computational analysis suggests a defect in NADH flux to the respiratory chain

We next utilized the computational model to investigate which molecular alterations could produce the observed respiratory phenotype. We simulated a standard OCR experiment (sequential addition of oligomycin, FCCP and antimycin A) by partial inhibition of the F1Fo ATP synthase, increase in the H+ leak and partial inhibition of complex III (Supporting Information Appendix S1). We then simulated impairments in individual modules by decreasing the activity of the relevant respiratory complex or increasing the activity of the H^+^ leak flux, and compared the predicted OCR to that obtained by the modelling of “wild‐type” conditions (WTsim, no simulated impairments). We considered an OCR metric to be altered if it was predicted to differ from WTsim by more than ±10%, and exceeded an additional threshold calculated using statistical power analysis. Somewhat surprisingly, the model predicted that defects in complex I (CI), CIII, CIV, F_1_F_o_ ATP synthase (baseline activity reduced to 70% of WTsim) or H^+^ leak (baseline activity increased to 150% of WTsim) would not recapitulate the behaviour observed in TgAD neurons (Figure 2d,e). In contrast, a simulated impairment in the NADH flux to the RC (baseline activity reduced to 95% of WTsim) was predicted to induce a strong decrease in maximal OCR, while leaving the other OCR metrics unchanged (Figure 2d,e,f), thereby recapitulating experimental measurements. Computational analysis therefore suggested that the reduction in maximal respiratory capacity measured in TgAD primary cortical neurons could not be explained by a defect in the RC, but rather by a defect in the provision of NADH (or other substrate) as input to the RC.

**Figure 3:**
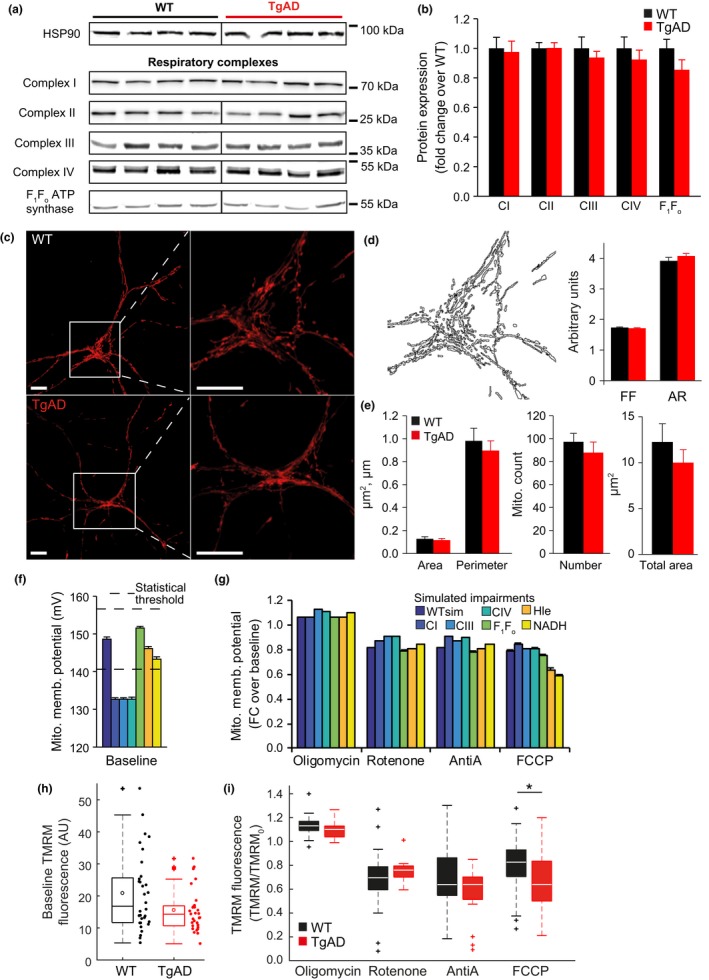
Primary cortical neurons from transgenic AD mice have preserved respiratory chain protein expression, mitochondrial morphology and network dynamics, while the mitochondrial membrane potential following uncoupling is significantly reduced. (a) Representative Western blots with molecular weight markings to the right of the blots, and (b) corresponding densitometry analysis of the expression level of subunits of the respiratory chain complexes (I‐IV) and the F_1_F_o_ ATP synthase, from WT and transgenic AD (TgAD) primary cortical neurons. The heat‐shock protein 90 (HSP90) was used as a loading control. *n* (independent cultures): WT 5 and TgAD 7. Black vertical lines indicate where some blots were cut to remove the molecular weight marker. (c) Representative confocal images of primary cortical neurons (after 6 DIV) transfected with a mitochondrial red fluorescent protein (mtDsRed) highlight the intricate mitochondrial network throughout the neuron. Scale bar, 10 μm. (d) Morphological characterization of the mitochondrial network after segmentation includes analysis of form factor (FF) and aspect ratio (AR), two geometric indicators of mitochondrial shape in terms of elongation and branching; and (e) measurements of the size (area and perimeter) and number of individual mitochondria, and the total mitochondrial area within individual neurons. *n* (independent cultures‐number of neurons): WT 3‐15, TgAD 3‐15; with an average of 80–100 mitochondria analysed per neuron. (f, g) Predicted mitochondrial membrane potential (f) at baseline (dashed lines indicate the statistical thresholds defined to identify whether predicted changes were likely to be measured experimentally [see Section 4]), and (g) fold change following addition of oligomycin (Oligo), rotenone, antimycin A (AntiA) or FCCP. Predictions were analysed with no simulated impairment (WTsim) and with impairments simulated in the indicated fluxes (100 simulations for each). Impairments were simulated by reducing fluxes to 70% of WTsim for respiratory complex I (CI), CIII, CIV and F_1_F_o_ ATP synthase; increasing the H^+^ leak flux (Hle) to 150% of WTsim; or reducing the NADH flux to 95% WTsim. (h) A significant difference in baseline TMRM fluorescence was not detected between WT and TgAD neurons (measured following addition of K+ gluconate to dissipate the plasma membrane potential). n: WT 4‐32, TgAD 2‐31. (i) No differences in TMRM fluorescence fold changes were measured between WT and TgAD neurons in response to F_1_F_o_ ATP synthase inhibition with oligomycin (2 μg/ml; n: WT 3‐21, TgAD 3‐16), complex I inhibition with rotenone (2 μM; n: WT 4‐17, TgAD 3‐16), nor complex III inhibition with antimycin A (AntiA 1 μM; n: WT 4‐20, TgAD 3‐21). In contrast, mitochondrial uncoupling with FCCP induced a significantly larger drop in TMRM fluorescence in TgAD neurons (n: WT 3‐44, TgAD 2‐18)

We next investigated whether the mitochondrial RC in our TgAD neurons was indeed intact. We measured the protein levels of respiratory complexes I, II, III and IV and the F_1_F_o_ ATP synthase, and indeed did not observe any significant difference between genotypes (Figure 3a,b). As measurements of mitochondrial activity could also be impacted by changes in the numbers of mitochondria or morphology of the network, we labelled mitochondria with a mitochondria‐targeted red fluorescent protein (mtDsRed; Figure 3c), and performed quantitative morphological analyses (Koopman et al., 2005). The form factor (FF) and aspect ratio (AR), two geometric indicators of mitochondrial shape in terms of elongation and branching, were similar in WT and TgAD neurons, suggesting unaltered mitochondrial dynamics (Figure 3d). Moreover, the size, number and total area of mitochondria were also similar in both genotypes (Figure 3e), collectively demonstrating intact mitochondrial integrity, morphology and network dynamics.

To further assess activity of the respiratory chain, we investigated mitochondrial membrane potential (ΔΨ_m_). The computational model predicted that, while impaired respiratory chain activity induces a measurable decrease in baseline ΔΨ_m_, a defect in NADH supply induces a smaller reduction (Figure 3f). The model also predicted that such an impairment has a minimal effect on ΔΨ_m_ fold changes in response to oligomycin, rotenone or antimycin A, but has a greater effect on the response to FCCP (Figure 3g). To investigate these predictions experimentally, we measured ΔΨ_m_ using TMRM. We first measured baseline ΔΨ_m_ by bathing neurons in a saline containing K‐gluconate to depolarize the plasma membrane and remove the confounding effect of plasma membrane potential changes on TMRM fluorescence (Tottene, Moretti, & Pietrobon, 1996; Ward et al., 2007). We did not measure a significant difference in baseline TMRM fluorescence between WT and TgAD neurons (Figure 3h). We next measured the TMRM fluorescence fold change in response to various mitochondrial inhibitors (Figure 3i). Addition of oligomycin, rotenone and antimycin A induced similar effects in both WT and TgAD neurons. In agreement with model predictions for a NADH defect however, mitochondrial uncoupling with FCCP induced a significantly stronger effect in TgAD neurons.

### 2.4 NAD(P)H autofluorescence measurements validate the computationally predicted mitochondrial NADH defect in transgenic AD neurons

**Figure 4:**
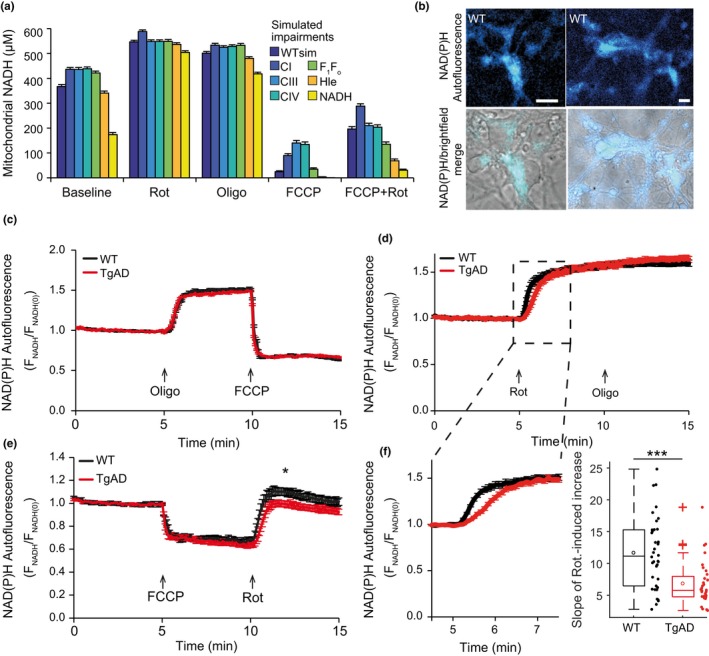
NAD(P)H autofluorescence measurements confirm the computationally predicted defect in mitochondrial NADH production in transgenic AD neurons. (a) Predicted mitochondrial NADH levels at baseline and following addition of rotenone (Rot), oligomycin (Oligo), FCCP and FCCP + rotenone (FCCP+Rot). NADH levels here represent redox status (NADH/NAD^+^), as we simulate a constant NADH size. Predictions were analysed as in Figure 3. (b) Representative NAD(P)H autofluorescence and brightfield images in WT primary cortical neurons. Scale bars, 10 μm. (c–e) Time‐series autofluorescence measurements (mean ± *SEM*) from the cell bodies of single neurons in WT and transgenic AD (TgAD) primary cortical neurons, normalized to the baseline signal. The times of drug additions are indicated with arrows. (c) No differences were seen between WT and TgAD neurons following induction of maximal NAD(P)H autofluorescence with oligomycin (Oligo; 2 μg/ml). Oligomycin indirectly inhibits NAD(P)H consumption by reducing respiratory chain activity. *n* (independent cultures‐number of neurons): WT 5‐37, TgAD 5‐38. (d) Similarly, NAD(P)H autofluorescence levels after rotenone (Rot; 2 μM) did not differ between WT and TgAD neurons. Rotenone directly blocks NAD(P)H consumption through its inhibition of complex I. n: WT 5‐38, TgAD 4‐32. (e) Minimal NAD(P)H autofluorescence levels following FCCP addition (0.5 μM) did not differ between genotypes, but levels following subsequent rotenone addition (Rot, 2 μM) were significantly lower in TgAD neurons compared to WT (*p < 0.05). n: WT 4‐20, TgAD 5‐27. (f) Average NAD(P)H autofluorescence time‐series traces enlarged from (d) (left) and rate of increase of autofluorescence signal following rotenone addition (right). The rate of increase was significantly slower in TgAD neurons (***p = 6 × 10^−5^)

We next utilized the computational model to suggest additional experiments that could further validate the presence of an impaired NADH supply to the RC. The model predicted that such an impairment significantly alters baseline mitochondrial redox status (NADH/NAD^+^) and the redox response to specific pharmacological inhibition (Figure 4a). While FCCP maximally increases RC activity and hence NADH consumption, this consumption is balanced by an increase in NADH production/import by mitochondria, thereby establishing a steady state. Thus, addition of rotenone (directly inhibiting NADH consumption by complex I) subsequent to FCCP will induce a rapid increase of NADH levels informative of mitochondrial NADH metabolism. In this instance, the model predicted that mitochondrial redox status, following the addition of FCCP plus rotenone, would be markedly reduced by an impairment in the NADH flux (Figure 4a). To validate these predictions, we measured NAD(P)H autofluorescence in our primary cultures, using classical epifluorescence microscopy (Figure 4b). We note that simulated redox status (WTsim) agrees with NAD(P)H autofluorescence measurements in WT cells exposed to oligomycin (Figure 4c), further demonstrating model agreement with intact WT neurons. Maximal and minimal NAD(P)H autofluorescence, as induced by oligomycin/rotenone and FCCP, respectively, were similar in WT and TgAD neurons (Figure 4c,d,e). In contrast, and in agreement with model predictions of an impaired mitochondrial NADH flux, NAD(P)H autofluorescence following FCCP plus rotenone addition was significantly lower in TgAD neurons compared to WT (−11%, *p* = 0.01, Figure 4e). Of note, we also observed that the rate of increase of autofluorescence signal following rotenone addition was significantly slower in TgAD neurons (Figure 4f; *p* = 6 × 10^−5^), further indicating a defect in mitochondrial NAD(P)H production/import. The model also predicted a reduction of basal NADH concentration (Figure 4a), a difference we did not detect via epifluorescence measurements. However, the low sensitivity of whole‐cell autofluorescence measurements performed by epifluorescence microscopy may prevent the detection of smaller differences at baseline. We therefore sought to undertake a more sensitive measure of NAD(P)H.

### 2.5 Transgenic AD neurons have reduced mitochondrial and glycolytic NAD(P)H concentration

**Figure 5:**
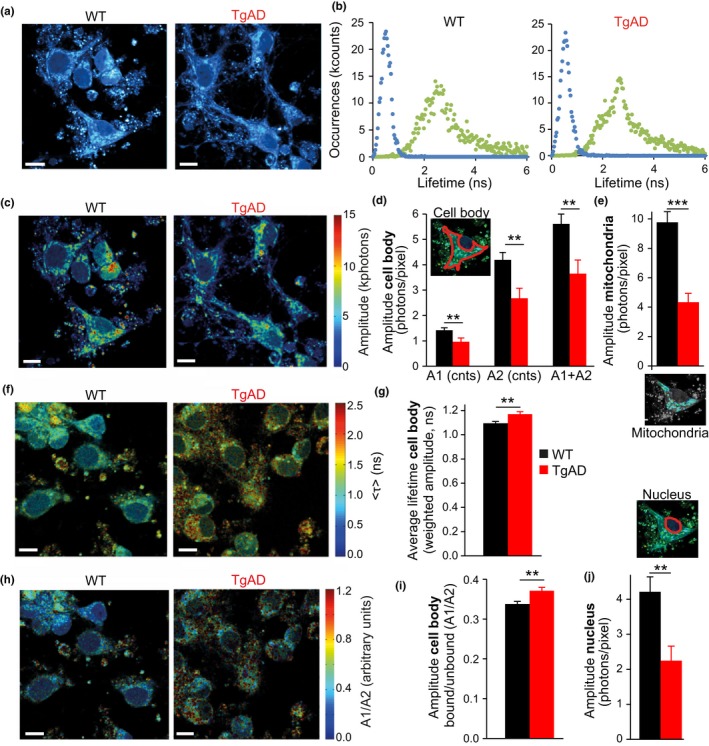
NAD(P)H FLIM measurements confirm reduced mitochondrial NAD(P)H in transgenic AD neurons and identify a reduction in cytosolic NAD(P)H. (a) Representative NAD(P)H autofluorescence intensity in primary cortical neurons (WT and TgAD), collected with a 2‐photon microscope under 740 nm excitation. (b) Distribution of photons as a function of their fluorescence lifetimes, illustrating the two NAD(P)H lifetimes—short (~0.5 ns, blue scatter points) and long (~2.8 ns, green scatter points)—corresponding respectively to free and protein‐bound NAD(P)H in WT and TgAD neurons. (c–j) FLIM measurements. The autofluorescence signal amplitude (d, e, j) is proportional to the NAD(P)H concentration at the specific lifetime. Average lifetimes weighted by amplitude (g) and ratio of the amplitudes at the long and short lifetimes (i) represent the proportion of aerobic/glycolytic metabolism of the neuron, in relation to NAD(P)H. **p < 0.01. (c) Representative images and (d) average amplitude of the NAD(P)H autofluorescence normalized by the ROI area in pixels, associated with the short (A1) and long (A2) lifetimes separated and together (A1+A2) detected in the cell body. *n* (independent cultures‐number of neurons): WT 5‐32, TgAD 4‐33. (e) Average total NAD(P)H amplitude in mitochondria. n: WT 5‐15, TgAD 4‐12. (f) Representative images and (g) average lifetime weighted by amplitude in WT and TgAD neurons in the cell body. (h) Representative images and (i) ratio of the amplitudes at the long and short lifetimes (A1/A2) in the cell body of WT and TgAD neurons. (j) Average amplitude of the NAD(P)H autofluorescence signal in the nucleus (equivalent to cytosol) of WT and TgAD neurons. For panels g, i and j, n: WT 5‐32, TgAD 4‐33. For all images, scale bar, 10 μm

To provide a more sensitive read‐out of NAD(P)H steady‐state levels, we next performed 2‐photon fluorescence lifetime imaging microscopy (FLIM) of NAD(P)H autofluorescence. The lifetime of fluorescence emitted following excitation of NAD(P)H is distributed into two populations, a short lifetime (~0.5 ns) associated with free NAD(P)H, and a longer lifetime (~2.8 ns) associated with protein‐bound, “active” NAD(P)H (Becker, 2015; Lakowicz, Szmacinski, Nowaczyk, & Johnson, 1992). FLIM can therefore measure the proportion of free and bound NAD(P)H (Vergen et al., 2012). Moreover, FLIM can also measure the overall amplitude of the NAD(P)H autofluorescence signal, independent of its lifetime, and is one of the most sensitive read‐outs available to measure NAD(P)H concentration in subcellular compartments of living cells, especially when combined with high spatial resolution and less toxic 2‐photon microscopy (Heikal, 2010; Figure 5a,b). Interestingly, the NAD(P)H autofluorescence signal amplitude (Figure 5c,d) in the cell body (excluding the nucleus) was significantly lower in TgAD neurons, both for protein‐bound long lifetime (A1, −32%, *p* = 0.01, Figure 5d) and free short lifetime (A2, −36%, *p* = 0.003, Figure 5d) forms. This could result in a general metabolic impairment, as indicated by significantly reduced total NAD(P)H levels in TgAD neurons (A1+A2, −35%, *p* = 0.005, Figure 5d). Using the spatial resolution of 2‐photon microscopy to isolate the signal directly in the mitochondria, we also measured a significant decrease in mitochondrial NAD(P)H in TgAD neurons (−50%, *p* = 0.003, Figure 5e). These data confirmed the predicted impairment in mitochondrial NAD(P)H homeostasis in TgAD neurons.

Importantly, FLIM measurements of NAD(P)H also enable investigation of the cellular metabolic phenotype. Indeed, empirical evidence has associated a shorter average lifetime, and a higher contribution of the short lifetime component, to a more glycolytic phenotype (Bird et al., 2005; Chakraborty, Nian, Tsai, Karmenyan, & Chiou, 2016; Schneckenburger, Wagner, Weber, Strauss, & Sailer, 2004; Skala et al., 2007). Intriguingly, we observed a higher average lifetime in TgAD neurons compared to WT (+7%, *p* = 0.006, Figure 5f,g), as well as a higher proportion of NAD(P)H molecules in the bound state (+10%, *p* = 0.003, Figure 5h,i), indicating that TgAD neurons were, proportionally, more aerobic compared to WT, despite lower mitochondrial NAD(P)H levels. As NAD(P)H freely diffuses across the nuclear membrane, the NAD(P)H signal amplitude in the nucleus represents cytosolic NAD(P)H levels (Aguilar‐Arnal et al., 2016; Blacker et al., 2014). Isolating the signal in the nucleus, we also measured a statistically significant reduction in NAD(P)H concentration in this compartment in TgAD neurons (−47%, *p* = 0.002, Figure 5j), indicating lower cytosolic NAD(P)H.

### 2.6 Transgenic AD neurons exhibit a glycolytic impairment

**Figure 6:**
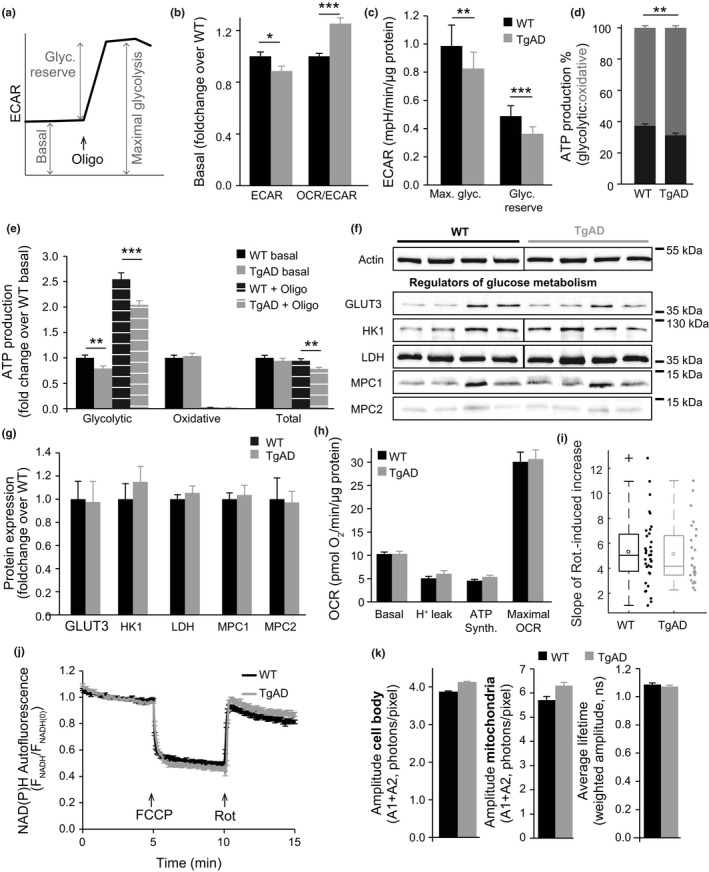
Extracellular acidification rate (ECAR) measurements demonstrate a glycolytic defect in transgenic AD neurons, and experiments in pyruvate suggest a causal relationship between glycolytic and mitochondrial impairments. (a) Experimental protocol followed to measure the extracellular acidification rate (ECAR) in primary cortical neurons. Maximal glycolysis = ECAR after inhibition of F_1_F_o_ ATP synthase activity with oligomycin (Oligo; 2 μg/ml). Glycolytic reserve (Glyc. reserve) = “spare” ECAR available while at rest (maximal–basal). (b) Basal ECAR and the oxygen consumption rate (OCR)/ECAR ratio in WT and transgenic AD (TgAD) neurons. TgAD neurons had significantly lower ECAR compared to WT, and were more aerobic (higher OCR/ECAR ratio). *p < 0.05, ***p < 0.001. *n* (independent cultures‐number of wells): WT 10‐134, TgAD 12‐120. (c) Maximal glycolysis (Max. glyc.) and glycolytic reserve were also significantly lower in TgAD transgenic neurons (**p < 0.01, ***p < 0.001). n: WT 5‐44, TgAD 7‐51. (d) Proportion of glycolytic (black) and oxidative (grey) ATP production in basal conditions. (e) Glycolytic, oxidative and total ATP production in basal conditions and following oligomycin addition (+Oligo; 2 μg/ml), normalized to WT basal levels. (d, e) **p < 0.01; ***p < 0.001. *n*: WT 10‐73, TgAD 12‐76. (f) Representative Western blots with molecular weight markings to the right of the blots, and (g) corresponding densitometry analysis of proteins involved in glucose metabolism—glucose transporter 3 (GLUT3; *n* (independent cultures): WT 5, TgAD 7), hexokinase 1 (HK1), lactate dehydrogenase (LDH), mitochondrial pyruvate carriers 1/2 (MPC1/2) (all *n*: WT 4, TgAD 4). Actin or HSP90 were used as loading controls. Black vertical lines indicate where some blots were cut to remove the molecular weight marker. (h–k) Measurements in WT and TgAD neurons supplemented with 5 mM pyruvate. (h) Basal OCR, proton leak, ATP synthesis and maximal OCR, as described in Figure 2a and measured by Seahorse (*n* = independent cultures‐number of wells: basal, WT 13‐107, TgAD 14‐110; H+ leak & ATP turnover, WT 7‐49, TgAD 7‐44; maximal, WT 6‐58, TgAD 7‐66). (i) Rate of increase of autofluorescence signal following rotenone (2 μM) addition (*n* = independent cultures‐number of neurons: WT 5‐35, TgAD 4‐26). (j) NAD(P)H autofluorescence following FCCP (0.5 μM) and rotenone (Rot, 2 μM) addition, as measured using epifluorescence microscopy (*n* = independent cultures‐number of neurons: WT 4‐25, TgAD 3‐25). (k) Amplitude of the NAD(P)H autofluorescence signal (A1+A2) and average lifetime in the cell body and in mitochondria, as measured by FLIM (*n* = independent cultures‐number of neurons: cell body, WT 5‐45, TgAD 6‐68; mitochondria, WT 5‐12, TgAD 6‐17)

Thus far, our findings argued against reduced respiratory complex activity in TgAD neurons. Moreover, reduced mitochondrial and cytosolic (nuclear) NAD(P)H levels measured by FLIM could be explained by an impaired glycolytic flux. We therefore analysed the extracellular acidification rate (ECAR), a read‐out for anaerobic glycolysis, using the Seahorse XF Analyzer (Figure 6a). Interestingly, in contrast to basal OCR measurements (Figure 2c), we found that basal ECAR was significantly lower in TgAD compared to WT neurons (–11%, *p* = 0.022, Figure 6b), suggesting an impaired glycolytic flux. Accordingly, and in further agreement with FLIM measurements, TgAD neurons exhibited a significantly higher OCR/ECAR ratio (+25%, *p* = 4 × 10^−7^, Figure 6b), indicating a more aerobic metabolic phenotype in the resting state (Kramer, Ravi, Chacko, Johnson, & Darley‐Usmar, 2014). The addition of oligomycin, by inhibiting mitochondrial ATP production, induces maximal glycolysis (Figure 6a). Maximal glycolysis and glycolytic reserve were also significantly lower in TgAD neurons (−16% and −26%, *p* = 0.004 and *p* = 2 × 10^−4^, respectively, Figure 6c).

We combined OCR and ECAR measurements to calculate the contribution of glycolysis and mitochondrial oxidative phosphorylation to ATP production rates (Mookerjee & Brand, 2015; Mookerjee, Gerencser, Nicholls, & Brand, 2017). Consistent with our previous data (Figure 5j), TgAD neurons showed reduced glycolytic ATP production both in the basal state and following oligomycin addition (−21% and −20%, *p* = 0.007 and 0.001, respectively, Figure 6e). Total ATP production was also lower in TgAD neurons following oligomycin (−17%, *p* = 0.003, Figure 6d), further suggesting impaired glycolysis in these neurons. Although total ATP production did not significantly differ between the two genotypes in the basal state (Figure 6e), the contribution of mitochondrial oxidative phosphorylation to ATP production was nevertheless higher in TgAD neurons, again indicating a more aerobic basal metabolism (+6%, *p* = 0.001, Figure 6d).

To investigate the source of glycolytic impairment, we measured the expression levels of several rate‐limiting enzymes and transporters associated with glucose metabolism—glucose transporter 3 (GLUT3), hexokinase 1 (HK1), lactate dehydrogenase (LDH) and mitochondrial pyruvate carriers 1 and 2 (MPC1‐2). Interestingly, we did not observe any differences between the two genotypes (Figure 6f,g), suggesting that a defect affecting glycolysis might originate from levels of regulation other than the expression levels of these proteins.

### 2.7 Reduced mitochondrial NAD(P)H in transgenic AD neurons is caused by an impaired glycolytic flux

We further investigated the causal relationship between the glycolytic impairment and mitochondrial NAD(P)H flux reduction. In TgAD neurons, an impaired glycolytic flux, and the resultant reduction in cytosolic NAD(P)H and carbon fluxes to mitochondria, could lead to decreased import/production of NAD(P)H by mitochondria. In this case, we hypothesized that providing pyruvate, rather than glucose, to the neurons would bypass any glycolytic impairment and restore mitochondrial health. We therefore repeated key experiments in which we observed significant differences compared to WT neurons, supplementing neurons with pyruvate (5 mM) rather than glucose. Interestingly, this ablated all significant NAD(P)H impairments measured in TgAD neurons (Figure 6h–k). As bypassing glycolysis suppressed mitochondrial defects in TgAD neurons, this indicated that a glycolytic defect may be primarily responsible for the mitochondrial NAD(P)H dyshomeostasis observed in these cells.

## 3 DISCUSSION

In this study, we utilized an interdisciplinary experimental and computational approach and found that primary cortical neurons from a transgenic AD mouse model have impaired glycolytic flux, reduced cytosolic and mitochondrial NAD(P)H concentrations, and defective mitochondrial respiratory capacity. Interestingly, the reduction of basal mitochondrial NAD(P)H was not associated with an impairment of basal respiration, indicating NAD(P)H levels are not a limiting factor for neuronal mitochondrial bioenergetics in our system (in such instances, compensatory mechanisms could bolster basal respiration, such as increased substrate supply to complex II). We postulate that the glycolytic defect may be primarily responsible for the observed phenotype, as bypassing glycolysis by supplementing neurons with pyruvate suppressed all mitochondrial defects. Mechanistically, an impaired glycolytic flux would lead to reduced cytosolic NADH production and reduced supply of carbon fluxes to mitochondria. This, in turn, would contribute to reduced mitochondrial NADH concentration by impairing both Krebs cycle activity and NADH transport into mitochondria via the malate–aspartate shuttle. The reduction of mitochondrial NADH would impair mitochondrial respiratory capacity, as predicted by the model and measured by experiments. The link between glycolysis and maximal respiration has been previously reported—inhibition of glycolysis by glucose restriction or pharmacological inhibitors can impair respiration generally (Clerc & Polster, 2012; Pike Winer & Wu, 2014; Zeidler et al., 2017), and maximal respiration specifically (Gouarne et al., 2013; Tan, Xiao, Li, Zeng, & Yin, 2015)—further validating the strong link between glucose metabolism and mitochondrial activity.

Both our experimental and computational analyses argued against a direct impairment of the mitochondrial RC in TgAD neurons. Although impairments in RC activity, specifically in complex IV, have been frequently associated with AD (Bosetti et al., 2002; Kish et al., 1992; ; Rhein et al., 2009; Yao et al., 2009), findings are not wholly consistent (Choi et al., 2012; Kipanyula et al., 2012), and studies were primarily performed in postmortem AD brains or in situations where clinical pathology was already apparent. In these instances, RC impairments may be related to Aβ toxicity (Pagani & Eckert, 2011). In our TgAD mice (B6.152H), elevated Aβ deposits have been detected in the brain at 3–6 months of age, with decreased complex IV activity and cognitive defects evident at 8 months (Ozmen et al., 2009; ; Rhein et al., 2009; Richards et al., 2003). Thus, it is noteworthy that the bioenergetic impairments reported here were identified in primary cortical neurons prepared from pups prior to any increased Aβ production/deposition or the onset of pathological behavioural phenotypes. These results further suggest a mechanism outside amyloid considerations for the bioenergetic impairment described here.

Our work, demonstrating glucose hypometabolism in vitro, echoes clinical research in humans, with glucose metabolism of increasing importance in AD research. Indeed, impaired glucose metabolism is one of the most reliable indicators of disease progression, potentially preceding clinical symptoms by years or even decades (Bubber et al., 2005; Chen & Zhong, 2013). However, the molecular mechanisms linking glycolysis defects to neurodegeneration remain elusive, with contrasting findings on the level of glucose metabolism enzymes and transporters in AD patients (Bigl, Bruckner, Arendt, Bigl, & Eschrich, 1999; Harr, Simonian, & Hyman, 1995), likely due to the fact that analyses were performed in patients with different pathologic phenotype onset. Further experimental or computational investigations are required to determine the precise molecular defects of this impairment. Interestingly, post‐translational O‐glycosylation may connect glucose hypometabolism and neurodegeneration. Glycosylation‐related enzymes are particularly abundant in neurons, and reduced glycolytic flux could impair glycosylation precursor biosynthetic pathways. Indeed, decreased O‐glycosylation has been observed in AD patients, its induction is protective in cell and animal models of AD (Zhu, Shan, Yuzwa, & Vocadlo, 2014), and inhibition of a glycoside hydrolase prevented cognitive decline, plaque formation and tau aggregates in transgenic mouse models of neurodegeneration (Hastings et al., 2017; Yuzwa et al., 2014). On this aspect, however, studies in appropriate neuronal models are lacking. Moreover, dysregulated Ca^2+^ signalling, as previously described in these TgAD neurons (Kipanyula et al., 2012), could also affect glucose/NAD(P)H metabolism (Llorente‐Folch et al., 2015; Pancani, Anderson, Porter, & Thibault, 2011), and Wnt signalling was recently suggested as a potential link between neuronal physiology, glucose metabolism and AD (Cisternas & Inestrosa, 2017).

Computational modelling provides an additional analytical technique enabling alternative investigations of complex cellular behaviour. Indeed, this study describes valuable experimental data interpretation and hypothesis generation explicitly facilitated by computational analyses. However, we do not claim that the model described here is a complete quantitative representation of the neuronal mitochondrial respiratory chain. Indeed, certain modelling predictions (e.g., the predicted relationship between basal and maximal respiration and respiratory complex impairment) warrant further investigations beyond the scope of this work. In any study, computational predictions should be carefully considered and thoroughly validated where possible. In our interdisciplinary approach, we first validated model predictions via both TMRM and NAD(P)H measurements, and further strengthened our hypothesis of a glycolytic defect by demonstrating the complete recovery of TgAD neurons when supplemented with pyruvate.

Our study highlights the powerful utility of an interdisciplinary, systems biology approach and clearly demonstrates that defects in glucose metabolism in vitro are indeed detectable in neurons prior to the onset of any sign of pathology in TgAD mice. This highlights potential for molecular investigations that could lead to new interventional approaches in the early diagnosis and treatment of AD.

## 4 EXPERIMENTAL PROCEDURES

### 4.1 Animals

All procedures were conducted in accordance with the Italian and European Communities Council Directive on Animal Care and were approved by the Italian Ministry of Health. Handling of animals was in accordance with Directive 2010/63/EU of the European Parliament on the protection of animals used for scientific purposes.

### 4.2 Primary cultures of cortical neurons

Primary neuronal cultures were obtained from cortices dissected from 0‐ to 1‐day newborn mice of either sex, as previously described (Kipanyula et al., 2012). Experiments were performed after 9–11 days in vitro (DIV) unless stated otherwise.

### 4.3 TMRM and NAD(P)H epifluorescence microscopy

Experiments were performed following the standardized protocols described recently (Connolly et al., 2017). After verifying signal stability, baseline measurements were used to normalize the traces. We utilized a combination of mitochondrial inhibitors to assess the impact on mitochondrial membrane potential (ΔΨ_m_) and NAD(P)H, and therefore investigate ΔΨ_m_ and NADH fluxes (Connolly et al., 2017). To measure baseline ΔΨ_m_, we bathed neurons in a saline containing 130 mM K‐gluconate to dissipate the plasma membrane potential (ΔΨ_p_) and overcome the confounding effect of baseline ΔΨ_p_ changes on TMRM fluorescence (Tottene et al., 1996; Ward et al., 2007). Drug concentrations are listed in Supporting Information Table S5.

### 4.4 Oxygraphy, extracellular acidification measurements and ATP calculations

Live OCR and extracellular acidification rate (ECAR) were measured simultaneously using a Seahorse XFe24 Analyzer (Agilent). Saline and equilibration conditions were the same as for live imaging. The classical “mitochondrial stress test” protocol was performed as previously described (Connolly et al., 2017). After calculation of the buffering power of the experimental saline and the proton rate production (Mookerjee & Brand, 2015), mitochondrial and cytosolic (glycolytic) ATP production rates were calculated from OCR and ECAR measurements as described previously (Mookerjee et al., 2017).

### 4.5 Immunofluorescence and transfection

Transfection of a mitochondria‐targeted red fluorescent protein DsRed (mtDsRed), cloned in a pcDNA3 backbone (Filadi et al., 2015), was performed the day after plating, 6 hr before the change from plating medium to growth medium, with Lipofectamine 2000 (Thermo Fisher), following the protocol provided by the manufacturer. Fixation and immunofluorescence were performed as previously described (Filadi et al., 2015). Primary antibodies directed against GFAP and NF200 are detailed in Supporting Information Table S6.

### 4.6 2‐photon NAD(P)H fluorescence lifetime imaging microscopy (FLIM)

Neurons were equilibrated at 37°C in the same saline as for NAD(P)H autofluorescence experiments, and imaging was performed at 22°C in the same saline with pH adjusted for temperature. FLIM measurements were carried out using the time‐correlated single‐photon counting (TCSPC) method. Amplitude (A1, A2) and fluorescence lifetime (τ_1_, τ_2_) parameters were extracted from the decay fitting.

### 4.7 Protein extraction and Western blotting

For extraction and denaturation of proteins, two different protocols were used: RC complexes, MPC1 and 2 and GLUT3 were extracted in RIPA buffer and denatured at 55°C; HK1 and LDH were extracted in Tris‐HCl and denatured at 70°C. After extraction, proteins were quantified using the BCA Protein Assay Kit (Pierce) following the manufacturer's instructions. Detection of proteins was performed by chemiluminescence. The intensity of the bands was analysed using the Fiji software program.

### 4.8 Morphological characterization of the mitochondrial network

Labelled mitochondria with a mitochondria‐targeted red fluorescent protein (mtDsRed) were morphologically analysed as described in Koopman et al. (2005) using the Fiji software (Schindelin et al., 2012).

### 4.9 Computational modelling

The core computational model was originally developed as a self‐contained thermodynamically balanced model of oxidative phosphorylation and the electron transport chain, based on experimental observations in isolated cardiac mitochondria (Beard, 2005). It has been compared to, and validated by, experimental data from in vitro cardiac and liver isolated mitochondria and in vivo skeletal muscle mitochondria (Bazil, Beard, & Vinnakota, 2016; Dash & Beard, 2008; Wu, Yang, Vinnakota, & Beard, 2007), and was extended (additional flux incorporated to describe cytosolic ATP processes) to analyse respiratory chain function in intact cancer cells (Huber, Connolly, Dussmann, & Prehn, 2012; Huber et al., 2011). We chose to implement this model (Figure 1a) to specifically focus our computational investigations on the mitochondrial respiratory chain, impairments of which have been extensively reported in AD (Bosetti et al., 2002; Kish et al., 1992; Rhein et al., 2009; Yao et al., 2009). To calibrate the model to intact primary neurons, parameter values (Supporting Information Tables S4) were either (a) obtained from peer‐reviewed publications, (b) retained from previous publications utilizing this model or (c) tuned to fit the model output to several in‐house experiments (Figure 1c,d). Initial concentrations (Supporting Information Tables S1) were maintained close to literature values where available. We simulated the addition of rotenone, antimycin A and oligomycin by reducing the kinetic constant of the flux through the relevant respiratory complex. FCCP addition was simulated by increasing the flux through the proton leak. Putative pathological impairments were similarly simulated, by increasing or reducing the kinetic constant of the flux through each complex. Full details are provided in the Supporting Information Appendix S1, and modelled state variables, initial concentrations, ordinary differential equations, fluxes, parameter values and relevant literature references are listed in Supporting Information Tables S1–S4. We implemented and analysed the model in MATLAB R2017a (The MathWorks, UK). Model code and the code to reproduce all figures have been deposited on GitHub (url: https://github.com/niamhconno/Theurey-et-al-2018).

### 4.10 Experimental design and statistical analysis

All n numbers are reported in the relevant figure legends. Bar charts show the mean ± standard error of the mean (*SEM*). Differences between means were determined by Student's *t* test. Boxplots show the lower, median and upper quartile values, and whiskers extend to the most extreme values within 1.5 times the interquartile range. Data points outside this range are marked by “+.” Differences between medians were determined using a rank sum test (MATLAB, The MathWorks). In all figures, * indicates *p* < 0.05, ** indicates *p* < 0.01, and *** indicates *p* < 0.001. To determine whether differences predicted by simulated impairments might be experimentally measured, we set a threshold using statistical power analysis (dashed lines in Figure 2d; see Supporting Information Appendix S1).

## CONFLICT OF INTEREST

None declared.

## AUTHORS’ CONTRIBUTIONS

PT, NMCC, PP and JHMP were responsible for experimental designs, data interpretation and writing of the paper. PT performed and analysed all the experiments. NMCC and SL parameterized the computational model and performed the simulations. PT and NMCC analysed and integrated experimental and modelling data. IF and CF performed and interpreted FLIM experiments. EM performed and analysed TMRM experiments. CMP, AJ, AG, DB, DP and MA participated in discussing the results. PP and JHMP provided funds.
