# Supplementary material for: Systems biology identifies preserved integrity but impaired metabolism of mitochondria due to a glycolytic defect in Alzheimer's disease neurons
Source: Aging Cell. 2019 Feb 21;18(3):e12924. doi: 10.1111/acel.12924 (PMC6516149; doi:10.1111/acel.12924)
Supplement: Supplementary file 1 [file ACEL-18-e12924-s001.docx]

**Extended Experimental Procedures**

Computational modelling

The computational model is a thermodynamically-balanced flux-based, ordinary differential equation model of the mitochondrial respiratory chain (RC) (Figure 1A). We implemented and analysed the model in MATLAB R2017a (The Mathworks, UK), and model code has been deposited on GitHub (url:<https://github.com/niamhconno/Theurey-et-al-2018>). The model structure has been extensively described elsewhere (Beard 2005, Huber *et al.* 2011, Huber *et al.* 2012). Briefly, the model comprises three compartments - a coarse-grain implementation of the cytosol, inter-membrane space and mitochondrial matrix, and 21 state variables, which include mitochondrial NADH, H^+^ and other ion concentrations, the mitochondrial membrane potential (ΔΨ_m_), and cytosolic/mitochondrial ATP/ADP (Supplementary table 1). Ordinary differential equations (ODEs, Supplementary table 2) mediate changes in these state variables, and are comprised of fluxes (J_*; Supplementary table 3). Parameter values are detailed in Supplementary table 4. These fluxes describe the activity of respiratory complexes I, III and IV, the F_1_F_o_ ATP synthase, proton (H^+^) leaks, the adenosine nucleotide transferase (ANT) and ion and H^+^ fluxes across the inner mitochondrial membrane. The supply of NADH as model input that drives the RC is described by a phosphate-dependent dehydrogenase flux (J_DH_), representing the requirement of phosphate as substrate for mitochondrial dehydrogenase enzymes (Beard 2005). Complex I and II are considered together and substrates for complex II (succinate, FAD) are embedded in the input dehydrogenase flux. As glucose metabolism produces 10 NADH but only 2 FAD molecules, this flux is dominated by NADH. Upstream bioenergetic processes, such as glycolytic flux, mitochondrial pyruvate transport, and TCA cycle flux, are considered embedded in this flux. The flux through CIII is described as a phosphate-dependent process, based on the hypothesis that phosphate modulates CIII activity (Beard 2005). Cytosolic ATP processes are described by a single flux (J_ATPK_) representing the ADP-dependent production of ATP and ATP-dependent consumption of ATP (Huber *et al.* 2011).

To simulate the cell-to-cell heterogeneity that may occur in any cell population, multiple simulations were run with parameter values and initial concentrations that varied within a normally distributed range of ±20% of the values listed in Supplementary tables 1 and 4 (mean = 0, s.d. = 10%). The variance of the simulated responses is lower than that of experimental measurements, most likely due to biological and technical variability not included in the model (such as the contribution of cytosolic processes, laser excitation fluctuations, etc.).

The simulated flux through complex IV serves as a proxy for mitochondrial O_2_ consumption. The simulated units (mol O_2_/s/litre of mitochondria) were converted to the experimentally measured mol O_2_/min/μg protein by assuming a mitochondrial volume of 4x10^-14^ l (Ward *et al.* 2007), and 300,000 neurons/well with 45 μg protein/well, as quantified in house. We therefore report oligomycin-sensitive and -insensitive respiration as proxies for ATP synthesis and H+ leak, respectively. We note that the real H+ leak flux may differ from the oligomycin-insensitive OCR – we chose to report these metrics to facilitate more direct comparison with experimental measurements in the majority of situations. We simulated the addition of rotenone, antimycin A and oligomycin by reducing the kinetic constant of, and therefore the flux through, the relevant respiratory complex (complex I (CI), CIII and the F_1_F_o_ ATP synthase, respectively). The extent of reduction was tuned to literature where respiratory complex activity was measured in synaptosomes exposed to varying concentrations of each inhibitor (Sipos *et al.* 2003; Telford *et al.* 2009), and to in-house measurements (Figure 1C,D). Therefore, 2 μM rotenone was simulated by reducing the flux through CI to 20% of its baseline flux, 1 μM Antimycin A was simulated by reducing the flux through CIII to 20% of its baseline flux, and 2 μg/ml oligomycin was simulated by reducing the flux through F_1_F_o_ to 13% of its baseline flux. When simulating antimycin A addition in OCR experiments, the fluxes through CI and CIII were set to ~0, to represent complete inhibition of the RC (any remaining OCR measured in experiments following rotenone/antimycin A addition is considered non-mitochondrial). For cell population simulations, simulated drug effects were varied between ⅓ and 3 times these values (normally distributed). FCCP addition was simulated by increasing the flux through the proton leak to 11.3*baseline flux, and varied between ±20% of this value for population simulations. To simulate putative pathological impairments in CI, CIII, CIV and F_1_F_o_ ATP synthase, we reduced the kinetic constant of the relevant flux (e.g. x_CI_ for CI; see Supplementary Table 3) to decrease the baseline flux to 70% of the flux simulated with no impairment (WTsim). An impairment in NADH flux was similarly simulated by a decrease in flux to 95% of WTsim. A H^+^ leak impairment was simulated by increasing the kinetic constant (x_Hle_) to increase the H^+^ leak flux to 150% of WTsim. Simulation of e.g. rotenone addition in the presence of a CI impairment therefore results in an additive effect on the flux through CI (with similar effects for other drugs/complex impairments).

Animals

The homozygous transgenic mouse line PS2APP (B6.152H line, PS2_N141I_×APP_swe_) was kindly donated by L. Ozmen and F. Hoffmann (La Roche Ltd, Basel, Switzerland) (Richards *et al.* 2003; Ozmen *et al.* 2009). The line has the background strain of C57BL⁄6 mice, which were used as wild type controls and purchased from Charles River (Lecco, Italy).

The mouse model is homozygous for PS2-N141I and APP Swedish (APPswe) K670N, M671L mutations, with the PS2 mutant ubiquitously expressed, and the APP mutant expressed only in neurons and thymocytes (Ozmen *et al.* 2009). TgAD mice produce similar levels of Aβ40 and Aβ42, and levels increase from the third to the sixth month of age, when the first amyloid deposits are detectable in the cortex and hippocampus (Richards *et al.* 2003; Ozmen *et al.* 2009). The first cognitive decline is detectable in these mice at eight months of age (Richards *et al.* 2003). In primary neuronal cultures (10-12 DIV) from transgenic neonatal pups (as used here), PS2 and APP levels are only twice that of controls, and total Aβ levels in brains of 2-week-old mice remain low, although detectable and higher in transgenic AD mice compared to WT (Kipanyula *et al.* 2012).

Primary cultures of cortical neurons

After removal of the hippocampus, cortices were dissociated in trypsin (1 mg/ml) for 10 min at 37°C and digestion was blocked with FCS 5% plus DNAse I (35 μg/ml). Cells were seeded on poly-L-lysine (100 μg.ml^-1^) coated coverslips at a density of 250,000 cells/cm^2^ for single cell imaging, or 1,000,000 cells/cm^2^ in Seahorse XF24 Cell Culture Microplates. The plating medium was MEM Gibco containing glucose (20 mM), L-glutamine (0.5 mM), N2 supplement (1%), B27 supplement (0.5%), biotin (3.6 μM), pyruvic acid (1 mM), penicillin (25 μg.ml^-1^), streptomycin (25 μg.ml^-1^), neomycin (50 μg.ml^-1^) and horse serum (10%). 24 h after plating, the plating medium was replaced with growth medium, composed of serum and antibiotic free Neurobasal medium containing glucose (25 mM), B27 (2%), L-glutamine (2 mM) and Cytosine-β- d-arabinofuranoside (3 μM). Fresh medium was added (1/4 of total volume) every 4 days. Unless otherwise stated, all materials were from Sigma Aldrich. The cellular composition of WT and transgenic AD primary cortical cultures (*i.e.,* percentage of astrocytes) was analysed by immunofluorescence using specific markers (neuron-specific NF200 and astrocyte-specific GFAP) on 10 DIV cultures. We did not observe any differences in the proportion of neurons and astrocytes. When performing single-cell experiments, neurons were morphologically identified as previously described (Abramov *et al.* 2007).

TMRM and NAD(P)H epifluorescence microscopy

Experiments were performed in a saline composed of 120 mM NaCl, 3.5 mM KCl, 0.4 mM KH_2_PO_4_, 5 mM NaHCO_3_, 20 mM HEPES, 1.2 mM Na_2_SO_4_, 1.2 mM CaCl_2_, 2 mM MgCl_2_, 10 mM glucose, pH 7.4 (NaOH). Before the experiments, neurons were equilibrated in the saline for 1 h at 37°C in the dark with no CO_2_ and with or without 10 nM tetramethylrhodamine methylester (TMRM). All experiments were performed at 37°C on an inverted microscope (Zeiss Axiovert 100) with a 40x oil objective (Fluar, NA1.30) and a cooled CCD camera (SensicamQE PCO, Kelheim, Germany). Excitation light was selected by a monochromator (polychrome V; TILL Photonics). TMRM was excited at 550 nm, through a Zeiss TRITC filter (emission 573-613 nm) and a dichroic mirror (565 FCXR). NAD(P)H autofluorescence was excited at 340 nm, through a neutral density filter (ND = 0.6), a 380 nm DLCLP dichroic and a bandpass filter D460/50. All filters and dichroics were from Chroma Technologies (Bellow Falls,VT, USA). Obtained images were then processed using the Fiji software (Schindelin *et al.* 2012). Imaging protocols were one acquisition/min with 50 ms exposure for TMRM, and one acquisition every 5 s with 750 ms exposure for NAD(P)H. Baseline TMRM and NAD(P)H (auto)fluorescence was recorded for 5-10 min. We utilised Rotenone and Antimycin A to inhibit complex I and complex III respectively, and oligomycin to inhibit the ATP synthase. Rotenone and oligomycin directly and indirectly inhibit mitochondrial NADH consumption by complex I. FCCP at low concentration (0.5 μM as determined by titration) uncouples respiration, driving uncontrolled NADH consumption by complex I and maximal RC activity. Drug concentrations are listed in Supplementary table 5.

We use the term ‘NAD(P)H’ when referring to experimental measurements, as these techniques do not distinguish between NADH and NADPH signals. As NADPH is not consumed by complex I, however, we use the term NADH when referring to fluxes to the RC.Given the high surface area of mitochondria within the cytoplasm of cortical neurons (Figure 3C), and the higher NAD(P)H levels in the mitochondria compared to those in the cytosol (Bilan *et al.* 2014), the overall NAD(P)H autofluorescence signal in the cell body of cortical neurons primarily reflects mitochondrial NAD(P)H (Shuttleworth 2010).

To measure baseline mitochondrial membrane potential (ΔΨ_m_) we modified the experimental saline to include 10 mM NaCl and 130 mM K^+^ gluconate, with 0 mM CaCl_2_. K^+^ gluconate dissipates the plasma membrane potential (ΔΨ_p_), minimising the contribution of ΔΨ_p_ to the TMRM signal (Ward *et al.* 2007; Tottene *et al.* 1996). 20 nM TMRM was used to compensate for the K^+^ gluconate-induced loss of ΔΨ_p_, and 5 images were obtained from each neuron.

TMRM and DiBAC imaging in neurons and relative quantification of mitochondrial membrane potential (Δψ_m_) for model calibration

Primary cortical neurons were obtained from embryonic (P16/17) WT 57BL/6 mice, under the license B100/4351 granted by the Irish Department of Health and Children with ethics approval from the RCSI (Research Ethics Committee #797), and cultured as described previously (D'Orsi *et al.* 2015). After 9-10 DIV, neurons were equilibrated with 10 nM tetramethylrhodamine methylester (TMRM, reporter of mitochondrial membrane potential) and 1 µM Bis-(1,3-Dibutylbarbituric Acid)Trimethine Oxonol (DiBAC, reporter of plasma membrane potential). Experimental saline and drug addition protocols were identical as for NAD(P)H epifluorescence experiments. Experiments were performed using both confocal and epifluorescence microscopy. We used a 40x oil-immersion objective lens on an inverted confocal laser-scanning microscope (LSM 510 Meta; Carl Zeiss), and excited TMRM with a 543 nm helium/neon laser (3%) and DiBAC with a 488 nm argon laser (1%). Emitted fluorescence was detected through a 560 nm long-pass filter (TMRM), and a 530-600 nm barrier filter (DiBAC). Epifluorescence imaging was performed on an Axiovert 200M inverted microscope equipped with a 40x, 1.4 numerical aperture oil-immersion objective (Zeiss), dichroic beam splitters, and filter wheels in the excitation and emission light path (TMRM: excitation, 530 ± 21 nm; emission, 592 ± 22 nm; DiBAC: excitation, 465 ± 25 nm; emission, 520 nm ± 25 nm). Images were processed using ImageJ and MetaMorph 7.5 (Molecular Devices). Relative Δψ_m_ and Δψ_p_ changes were quantified in MATLAB based on the Nernst equation (Ward *et al.* 2007), assuming baseline potentials of -147 mV (Δψ_m_, ref) and -83 mV (Δψ_p_) (Nicholls 2006). Results of experiments in neurons were then used for model calibration (Figure 1C).

Oxygraphy, extracellular acidification measurements and ATP calculations using a Seahorse XFe24 Analyzer

The classical ‘mito stress test’ protocol composed of subsequent additions of oligomycin, FCCP and antimycin A (Figure 2A) allows the analysis of the contribution of isolated components of the RC to the OCR (Connolly *et al.* 2017). Drug concentrations are listed in Supplementary table 5. The optimal FCCP concentration to induce maximal stable respiration was determined, after titration, to be 0.5 μM. After experiments, saline was removed, cells were lysed with 50 μL RIPA buffer per well (see RIPA composition in the Western Blotting section below), and total protein quantity was measured by BCA assay. This was used to normalise OCR and ECAR measurements between plates. The standard Seahorse protocol comprises three two-minute measurements between each injection. The average of the three measurements at each stage were used for analysis. We validated the specificity of the ECAR signal through a dedicated protocol using glucose, oligomycin to activate, and 2-deoxyglucose to inhibit glycolysis.

Immunofluorescence

Cells were washed with PBS, fixed with PFA (4%)–Saccarose (20%) for 10 min and quenched with 50 mM NH_4_Cl in PBS. For immunofluorescence, cells were then permeabilized for 4 min with 0.1% Triton X-100 in PBS and blocked in PBS containing 2% (wt/vol) BSA, 10% (vol/vol) goat serum and 0.2% gelatin for 1 h. Cells were then incubated with primary antibodies directed against GFAP and NF200 as detailed in Supplementary table 6 for 1 h at room temperature and were washed three times with the blocking solution. Alexa Fluor 488 and 555 conjugated secondary antibodies (Invitrogen) were diluted to 1/500 and applied for 45 min at room temperature. Finally, coverslips were washed three times with the blocking solution and once with bi-distilled water, and mounted with ProLong Gold Antifade reagent (Invitrogen). mtDSRed images were acquired on an upright Zeiss LSM700 confocal microscope with a 100x objective. Immunofluorescence was observed on an upright VIKO-Nikon pseudo-confocal system with a 10x objective.

2-photon NAD(P)H fluorescence lifetime imaging microscopy (FLIM)

Experiments were performed on an upright confocal laser scanning microscope (FV300/IX-71, Olympus), coupled with a PicoHarp 300 TCSPC electronics (PicoQuant) and a single-photon counting avalanche photodiode (SPAD, MPD, Italy). Two-photon excitation was provided by a Ti:Sapphire fs laser system (Coherent, Mira900-F, 76 MHz), tuned to 740 nm. The laser beam was focused onto the sample using a 60x 1.2NA water immersion microscope objective, with on-sample power maintained around 24 mW to reduce the specimen photodamage. The excitation beam was scanned continuously on the sample for 12 min with a pixel dwell time of 11 ms. A bandpass filter (470-495 nm) was used in order to detect only the NAD(P)H autofluorescence. Data acquisition was performed in reverse start-stop mode, with each detected photon assigned to one of the 406 channels with 32 ps width. Lifetime decays, extracted from the signals collected from a selected region of interest (ROI) containing the mitochondrial area of a single cell, were analyzed with the Symphotime software (SPT64-PicoQuant) using a two-component exponential model. Amplitude (A1, A2) and fluorescence lifetime (τ_1_, τ_2_) parameters were extracted from the decay fitting. Typical FWHM (full width at half maximum) of the instrumental response function (IRF), calculated with the IRF reconstruction script of the Symphotime software, was ~150-200 ps. FLIM analysis was then performed using a pixel-by-pixel 2-exponential reconvolution fit procedure. The average lifetime ($\left\langle\tau\right\rangle$) for each pixel or for single ROIs was calculated using the following formula:

$$\left\langle\tau\right\rangle=\frac{A1\cdot\tau_{1}+A2\cdot\tau_{2}}{A1+A2}$$

The distribution of lifetimes (Figure 5B) and values of lifetime and relative amplitudes (data not shown) measured in the different cellular compartments in our acquisitions were consistent with literature (Heikal 2010; Bilan *et al.* 2014; Blacker *et al.* 2014; Aguilar-Arnal *et al.* 2016), validating signal specificity.

Protein extraction and Western Blotting

For protein extraction, 2x10^6^ cells were solubilized in RIPA buffer (50 mM Tris, 150 mM NaCl, 1% Nonidet P-40, 0.5% deoxycholic acid, 0.1% SDS, pH 7.5) or in 30 mM tris-HCl pH8, with protease and phosphatase inhibitor mixtures (Roche), and incubated on ice for 30 min. Unsolubilized material was spun down at 10,000 × g for 10 min at 4 °C. After 10 min of temperature denaturation, proteins were separated by SDS–PAGE, transferred onto nitrocellulose membranes (GE Healthcare), and probed using various antibodies (Supplementary table 6). For RC complexes, MPC1&2 and GLUT3, 35 μg protein/lane was loaded on the gel; for HK1 and LDH, 20 μg protein/lane was loaded. Actin and Hsp90 protein levels were used as loading controls. Isotype-matched, HRP-conjugated secondary antibodies (Bio-Rad) were for detection (GE Healthcare).

Morphological characterisation of the mitochondrial network

Briefly, the background was subtracted, and a convolve filter and a mask were applied. Using the software’s analyse particle function, the form factor (FF) and aspect ratio (AR), two geometric indicators of mitochondrial shape in terms of elongation and branching, were calculated to assess mitochondrial morphology. Results are expressed as the average of values per cell profile.

Experimental design and statistical analysis

To determine whether differences predicted by simulated impairments might be experimentally measured, we set a threshold using statistical power analysis, using the sampsi function in Stata 12.1 (StataCorp, Texas, USA). We calculated the mean difference required for each metric to reach statistical significance (α < 0.05) at a power of 0.8, based on the number of neurons/wells (n), and the mean and standard deviation from all experiments. Any predicted differences that do not exceed this threshold may be difficult to measure experimentally.

**Extended tables**

**Table S1: Modelled state variables, their initial concentrations (following steady-state calculations) and literature references where available.**

| **State Variable** | **Description** | **Initial Conc.** | **Unit** | **Reference** |
| --- | --- | --- | --- | --- |
| H_x_ | Proton (H^+^; matrix) | 1.8x10^-8^ | M | pH = 7.7; (Bolshakov et al., 2008) |
| K_x_ | Potassium (K^+^; matrix) | 55 | mM |  |
| Mg_x_ | Magnesium (Mg^2+^; matrix) | 400 | μM |  |
| NADH_x_ | NADH (matrix) | 350 | μM | (Wei et al., 2011) |
| QH_2_ | Ubiquinol (matrix) | 40 | μM |  |
| C_red_ | Reduced cytochrome-*c* | 180 | μM |  |
| ATP_x_ | Total ATP (matrix) | 1.1 | mM | (Yoshida et al., 2016) |
| ADP_x_ | Total ADP (matrix) | 1.5 | mM |  |
| ATP_mx_ | ATP bound to Mg^2+^ (matrix) | 730 | μM |  |
| ADP_mx_ | ADP bound to Mg^2+^ (matrix) | 830 | μM |  |
| Pi_x_ | Inorganic phosphate (matrix) | 16 | mM |  |
| ATP_i_ | Total ATP (IMS) | 2.3 | mM |  |
| ADP_i_ | Total ADP (IMS) | 290 | μM |  |
| AMP_i_ | Total AMP (IMS) | 0 | M |  |
| ATP_mi_ | ATP bound to Mg^2+^ (IMS) | 2.3 | mM |  |
| ADP_mi_ | ADP bound to Mg^2+^ (IMS) | 290 | μM |  |
| Pi_i_ | Inorganic phosphate (IMS) | 20 | mM |  |
| dPsi | Mitochondrial membrane potential (ψΔ_m_) | 149 | mV | (Nicholls and Budd, 2000; Ward et al., 2000; Gerencser et al., 2012) |
| H_i_ | Hydrogen (H^+^, protons; IMS) | 3.9x10^-8^ | M |  |
| ATP_c_ | ATP (cytosol) | 2.3 | mM | (Nelson et al., 2008) |
| ADP_c_ | ADP (cytosol) | 290 | μM | ATP_c_/ADP_c_ ≈ 10; (Katsura et al., 1993; Hardie and Hawley, 2001) |

*Suffixes: _x_, mitochondrial matrix; _i_, mitochondrial intermembrane space (IMS); _c_, cytosolic; _m_, Mg^2+^ bound; _red_, reduced; _tot_, total*

**Table S2: Ordinary differential equations (ODEs) describing the state variables listed in Table 1-1. Fluxes (J_*) and parameters contributing to the ODEs are described in Tables 1-3 and 1-4. Detailed explanation of these ODEs can be found in (Beard, 2005; Huber et al., 2011).**

| **State Variable** | **Ordinary Differential Equation** | **Note** |
| --- | --- | --- |
| H_x_ | $\frac{x_{buff} * H_{x} * (+1*J_{DH} - (4+1)*J_{C1} - (4-2)*J_{C3} - (2+2)*J_{C4} + (n_{A}-1)*J_{F1} + 2*J_{Pi1} + J_{Hle} - J_{KH})}{W_{x}}$ |  |
| K_x_ | $\frac{J_{KH} + J_{K}}{W_{x}}$ |  |
| Mg_x_ | $\frac{{-J_{MgATP}}_{x}{-J_{MgADP}}_{x}}{W_{x}}$ |  |
| NADH_x_ | $\frac{J_{DH} - J_{C1}}{W_{x}}$ |  |
| QH_2_ | $\frac{J_{C1} - J_{C3}}{W_{x}}$ | 1 |
| C_red_ | $\frac{2J_{C3} - 2J_{C4}}{W_{i}}$ |  |
| ATP_x_ | $\frac{J_{F1} - J_{ANT}}{W_{x}}$ |  |
| ADP_x_ | $\frac{-J_{F1} + J_{ANT}}{W_{x}}$ |  |
| ATP_mx_ | $\frac{{J_{MgATP}}_{x}}{W_{x}}$ |  |
| ADP_mx_ | $\frac{{J_{MgADP}}_{x}}{W_{x}}$ |  |
| Pi_x_ | $\frac{-J_{F1} + J_{Pi1}}{W_{x}}$ |  |
| ATP_i_ | $\frac{J_{ATP} +J_{ANT} + {J_{AK}}_{i}}{W_{i}}$ |  |
| ADP_i_ | $\frac{J_{ADP} -J_{ANT} - 2{J_{AK}}_{i}}{W_{i}}$ |  |
| AMP_i_ | $\frac{J_{AMP} + {J_{AK}}_{i}}{W_{i}}$ |  |
| ATP_mi_ | $\frac{{J_{MgATP}}_{i}}{W_{i}}$ |  |
| ADP_mi_ | $\frac{{J_{MgADP}}_{i}}{W_{i}}$ |  |
| Pi_i_ | $\frac{-J_{Pi1} + J_{Pi2}}{W_{i}}$ |  |
| dPsi | $\frac{4J_{C1} + 2J_{C3} + 4J_{C4} - n_{A}*J_{F1} - J_{\mathrm{ANT}} - J_{\mathrm{Hle}} - J_{K}}{C_{\mathrm{IM}}}$ |  |
| H_i_ | $\frac{-J_{DH} + (4+1)*J_{C1} + (4-2)*J_{C3} + (2+2)*J_{C4} - (n_{A}-1)*J_{F1} - 2*J_{Pi1} - J_{Hle} + J_{KH} + J_{Ht})}{W_{i}}$ |  |
| ATP_c_ | $\frac{-J_{ATP} - J_{ATPK}}{W_{c}}$ |  |
| ADP_c_ | $\frac{-J_{ADP} + J_{ATPK}}{W_{c}}$ |  |

*Suffixes: _x_, mitochondrial matrix; _i_, mitochondrial intermembrane space (IMS); _c_, cytosolic; _m_, Mg^2+^ bound; _red_, reduced; _tot_, total*

*^1^As the inner mitochondrial membrane is not explicitly modelled as a separate compartment, the model describes the binding of QH_2_ to the respiratory complexes without specifying the precise location of this event. QH_2_ concentration is simulated as the concentration in the mitochondrial matrix*

**Table S3: Fluxes contributing to the ODEs described in Table 1-2, as described previously (Beard, 2005). Parameters contributing to these fluxes are described in Table 1-4.**

| **Flux** | **Description** | **Equation** | **Unit** |
| --- | --- | --- | --- |
| Membrane proton-motive forces and respiration fluxes | | |  |
| dG_H_ | Protomotive force | $F*dPsi+1*RT*\log\left( \frac{H_{i}}{H_{x}} \right)$ |  |
| dG_C1op_ | Gibb’s energy complex I | $dG_{C1o}-1*RT*log(\frac{H_{x}}{1e^{-7}})$ |  |
| dG_C3op_ | Gibb’s energy complex III | $dG_{C3o}+2*RT*log(\frac{H_{x}}{1e^{-7}})$ |  |
| dG_C4op_ | Gibb’s energy complex IV | $dG_{C4o}-2*RT*log(\frac{H_{x}}{1e^{-7}})$ |  |
| dG_F1op_ | Gibb’s energy F_1_F_o_ ATP synthase | $dG_{F1o}-1*RT*log(\frac{H_{x}}{1e^{-7}})$ |  |
| J_DH_ | Mitochondrial dehydrogenase flux (input NADH flux) | $x_{DH}*\left( r_{DH}*NAD_{x}-NADH_{x} \right)*\frac{1+{Pi_{x}}/{k_{Pi1}}}{1+{Pi_{x}}/{k_{Pi2}}}$ |  |
| J_C1_ | Flux through complex I | $x_{c1}*\left( \exp\left( \frac{-\left( dG_{C1op}+4dG_{H} \right)}{RT} \right)*NA{DH}_{x}*Q-NAD_{x}*QH_{2} \right)$ | mol s^-1^ (l mito)^-1^ |
| J_C3_ | Flux through complex III | $x_{c3}*\frac{1+{Pi_{x}}/{k_{Pi3}}}{1+{Pi_{x}}/{k_{Pi4}}} *\exp\left( \frac{-\left( dG_{C3op}+4dG_{H}-2F*dPsi \right)}{2RT} \right)*C_{ox}*\surd QH_{2}-C_{red}*\surd Q$ | mol s^-1^ (l mito)^-1^ |
| J_C4_ | Flux through complex IV | $x_{c4}*\frac{O_{2}}{O_{2}+k_{O2}} *\frac{C_{red}}{C_{tot}}\exp\left( \frac{-\left( dG_{C4op}+2dG_{H} \right)}{2RT} \right)*C_{red}*O_{2}^{0.25}-C_{ox}*exp(F*\frac{dPsi}{RT})$ | mol s^-1^ (l mito)^-1^ |
| J_F1_ | Flux through F_1_F_o_ ATP synthase | $x_{F1}*\left( \exp\left( \frac{-\left( dG_{F1op}-n_{A}*dG_{H} \right)}{RT} \right)*\frac{K_{DD}}{K_{DT}}*ADP_{mx}*Pi_{x}-ATP_{mx} \right)$ | mol s^-1^ (l mito)^-1^ |
|  |  |  |  |
| ATP transferase | | |  |
| J_ANT_ | Adenosine nucleotide transferase | $x_{ANT}*\left( \frac{ADP_{fi}}{ADP_{fi}+ATP_{fi}*\exp\left( -F*\frac{Psi_{i}}{RT} \right)}-\frac{ADP_{fx}}{ADP_{fx}+ATP_{fx}*\exp\left( -F*\frac{Psi_{x}}{RT} \right)} \right)* \frac{{ADP}_{fi}}{ADP_{fi}+k_{mADP}}$ | mol s^-1^ (l mito)^-1^ |
|  |  |  |  |
| Ionic Fluxes |  |  |  |
| H_2_Pi_i_ | Pi H^+^ binding (IMS) | $Pi_{i}*H_{i}*\left( H_{i}+k_{dHPi} \right)$ |  |
| H_2_Pi_x_ | Pi H^+^ binding (matrix) | $Pi_{x}*\frac{H_{x}}{\left( H_{x}+k_{dHPi} \right)}$ |  |
| J_Pi1_ | Pi/H^+^ exchanger | $x_{Pi1}*\frac{H_{x}*H_{2}{Pi}_{i}-H_{i}*H_{2}{Pi}_{x}}{H_{2}Pi_{i}+k_{PiH}}$ | mol s^-1^ (l mito)^-1^ |
| J_Hle_ | Proton (H^+^) leaks | $x_{Hle}*dPsi*\frac{H_{i}*exp(F*\frac{dPsi}{RT})-H_{x}}{\exp\left( F*\frac{dPsi}{RT} \right)-1}$ | mol s^-1^ (l mito)^-1^ |
| J_KH_ | K^+^/ H^+^ exchanger | $x_{KH}*\left( K_{i}*H_{x}-K_{x}*H_{i} \right)$ | mol s^-1^ (l mito)^-1^ |
| J_K_ | K^+^ uniport | $x_{K}*dPsi*\frac{K_{i}*\exp\left( F*\frac{dPsi}{RT} \right)-K_{x}}{\exp\left( F*\frac{dPsi}{RT} \right)-1}$ |  |
| J_AKi_ | Mitochondrial adenylate kinase | $x_{AK}*\left( K_{AK}*ADP_{i}*ADP_{i}-AMP_{i}*ATP_{i} \right)$ | mol s^-1^ (l mito)^-1^ |
| J_AMP_ | AMP MOM permeability | $gamma*x_{A}*\left( AMP_{e}-AMP_{i} \right)$ | mol s^-1^ (l mito)^-1^ |
| J_ADP_ | ADP MOM permeability | $gamma*x_{A}*\left( ADP_{e}-ADP_{i} \right)$ | mol s^-1^ (l mito)^-1^ |
| J_ATP_ | ATP MOM permeability | $gamma*x_{A}*\left( ATP_{e}-ATP_{i} \right)$ | mol s^-1^ (l mito)^-1^ |
| J_Pi2_ | Phosphate MOM permeability | $gamma*x_{Pi2}*\left( {Pi}_{e}-{Pi}_{i} \right)$ |  |
| J_Ht_ | Proton MOM permeability | $gamma*x_{Ht}*\left( H_{e}-H_{i} \right)$ |  |
|  |  |  |  |
| J_MgATPx_ | Mg^2+^ binding to ATP_x_ | $x_{MgA}*\left( ATP_{fx}*Mg_{x}-K_{DT}*ATP_{mx} \right)$ |  |
| J_MgADPx_ | Mg^2+^ binding to ADP_x_ | $x_{MgA}*\left( ADP_{fx}*Mg_{x}-K_{DD}*ADP_{mx} \right)$ |  |
| J_MgATPi_ | Mg^2+^ binding to ATPi | $x_{MgA}*\left( ATP_{fi}*Mg_{i}-K_{DT}*ATP_{mi} \right)$ |  |
| J_MgADPi_ | Mg^2+^ binding to ADPi | $x_{MgA}*\left( ADP_{fi}*Mg_{i}-K_{DD}*ADP_{mi} \right)$ |  |
|  |  |  |  |
| Cytosolic Energy balance to model intact cells | | |  |
| J_ATPK_ | Cytosolic ATP processes | $x_{ATPK}*\left( ATP_{e}-K_{ADTP\_dyn}*ADP_{e} \right)$ |  |

*Suffixes: x, mitochondrial matrix; i, mitochondrial intermembrane space (IMS); c, cytosolic; e, cytosolic (external); m, Mg^2+^ bound; red, reduced; tot, total*

*Abbreviations: MOM, mitochondrial outer membrane; Pi, phosphate*

**Table S4: Model parameters, their calibrated values, and literature references where available**

| **Symbol** | **Description** | **Value** | **Unit** | |  | **Reference** |  |
| --- | --- | --- | --- | --- | --- | --- | --- |
| NAD_tot_ | Total NAD(H) concentration (NAD^+^+NADH) | 726x10^-6^ | M | | c) | (Cloutier et al., 2009; Wei et al., 2011) |  |
| C_tot_ | Total cytochrome-*c* concentration (C_red_+C_ox_) | 2.7x10^-3^ | M | | b) | (Beard, 2005) |  |
| Q_tot_ | Total ubiquinol/ubiquinone concentration (Q+QH_2_) | 1.4x10^-3^ | M | | b) | (Dash and Beard, 2008) |  |
| ADTP_tot_ | Total cytosolic adenosine phosphates | 2.6x10^-3^ | M | | a) | (Cloutier et al., 2009; DiNuzzo et al., 2010) |  |
| O_2_ | Oxygen concentration | 26x10^-6^ | M | | a) | (Aubert and Costalat, 2005; Murphy, 2009; Dmitriev et al., 2015) |  |
| Dehydrogenase flux input function | |  |  | |  |  |  |
| k_Pi1_ | Dehydrogenase flux input (phosphate dependency) | 0.13x10^-3^ | M | | b) | (Beard, 2005) |  |
| k_Pi2_ | Dehydrogenase flux input (phosphate dependency) | 0.68x10^-3^ | M | | b) | (Beard, 2005) |  |
| x_DH_ | Dehydrogenase activity (flux activity) | 59 x10^-3^ | mol s^-1^ M^-1^ (l mito vol)^-1^ | | c) | (Dash and Beard, 2008) |  |
| r_DH_ | NADH/NAD^+^ equilibrium constant | 4.3 | Unitless | | c) | (Beard, 2005) |  |
|  |  |  |  | |  |  |  |
| Complex I | |  |  | |  |  |  |
| x_C1_ | Complex I activity | 1020 | mol s^-1^ M^-2^ (l mito vol)^-1^ | | b) | (Huber et al., 2012) |  |
|  |  |  |  | |  |  |  |
| Complex III | |  |  | |  |  |  |
| x_C3_ | Complex III activity | 224 x10^-3^ | mol s^-1^ M^-3/2^ (l mito vol)^-1^ | | b) | (Huber et al., 2012) |  |
| k_Pi3_ | Pi dependency parameter 1 | 0.19x10^-3^ | M | | b) | (Beard, 2005) |  |
| k_Pi4_ | Pi dependency parameter 2 | 25x10^-3^ | M | | b) | (Beard, 2005) |  |
|  |  |  |  | |  |  |  |
| Complex IV | |  |  | |  |  |  |
| x_C4_ | Complex IV activity | 0.32 x10^-3^ | mol s^-1^ M^-1^ (l mito vol)^-1^ | | b) | (Huber et al., 2012) |  |
| k_O2_ | Saturation constant for oxygen consumption | 0.12 x10^-3^ | M | | b) | (Dash and Beard, 2008) |  |
|  |  |  |  | |  |  |  |
| ATP synthase | |  |  | |  |  |  |
| x_F1_ | F_1_F_o_ ATP synthase activity | 6829 | mol s^-1^ M^-1^ (l mito vol)^-1^ | | b) | (Huber et al., 2012) |  |
|  |  |  |  | |  |  |  |
| Mg-binding to ATP/ADP | |  |  | |  |  |  |
| K_DT_ | Mg^2+^/ATP binding constant | 192x10^-6^ | M | | c) |  |  |
| K_DD_ | Mg^2+^/ADP binding constant | 347 x10^-6^ | M | | b) | (Dash and Beard, 2008) |  |
| x_MgA_ | Mg^2+^ binding activity | 1 x10^6^ |  | | b) | (Beard, 2005) | |
|  |  |  |  | |  |  |  |
| Cytosolic ATP production and consumption | |  |  | |  |  |  |
| x_ATPK_ | Cytosolic ATP production & consumption activity | 0.5 | min^-1^ | | c) |  |  |
| K_ADTP_dyn_ | ATP consumption equilibrium constant | 3.4 | Unitless | | c) |  |  |
|  |  |  |  | |  |  |  |
| Adenosine transferase | |  |  | |  |  |  |
| x_ANT_ | ANT activity | 2 x10^-3^ | mol s^-1^ (l mito vol)^-1^ | | b) | (Huber et al., 2011) |  |
| k_mADP_ | ANT parameter 1 | 3.5 x10^-6^ | M | | b) | (Dash and Beard, 2008) |  |
|  |  |  |  | |  |  |  |
| Proton leaks | |  |  | |  |  |  |
| x_Hle_ | Proton leak activity | 150 | mol s^-1^ M^-1^ mV^-1^ (l mito vol)^-1^ | | b) | (Dash and Beard, 2008; Huber et al., 2011) |  |
| OM transporters | |  |  | |  |  |  |
| x_Ht_ | MOM permeability to protons | 2000 | μm s^-1^ | | a) | ^2^ |  |
| gamma | MOM area per unit (mito) volume | 5.99 | μm^-1^ | | b) | (Beard, 2005) |  |
| x_A_ | MOM permeability to nucleotides | 85 | μm s^-1^ (l mito)^-1^ | | b) | (Dash and Beard, 2008) |  |
| x_Pi2_ | MOM permeability to phosphate | 327 | μm s^-1^ (l mito)^-1^ | | b) | (Dash and Beard, 2008) |  |
|  |  |  |  | |  |  |  |
| Phosphate-hydrogen co-transport | |  |  | |  |  |  |
| k_dHPi_ | H^+^/Pi co-transport binding constant | 10^-6.75^ | M | | b) | (Dash and Beard, 2008) |  |
| k_PiH_ | H^+^/Pi co-transport dissociation constant | 0.45 x10^-3^ | M | | b) | (Beard, 2005) |  |
| x_Pi1_ | H^+^/Pi co-transport activity | 385 x10^3^ | mol s^-1^ M^-1^ (l mito vol)^-1^ | | b) | (Dash and Beard, 2008) |  |
|  |  |  |  | |  |  |  |
| Potassium-hydrogen anti-port | |  |  | |  |  |  |
| x_KH_ | K^+^/H^+^ antiporter activity | 29.8 x10^6^ | mol s^-1^ M^-2^ (l mito vol)^-1^ | | b) | (Beard, 2005) |  |
|  |  |  |  | |  |  |  |
| Membrane buffer and capacitance | |  |  | |  |  |  |
| x_buff_ | Inner matrix H^+^ buffer capacity | 100 | M^-1^ | | b) | (Beard, 2005) |  |
| C_IM_ | Mitochondrial inner membrane capacitance | 6.8 x10^-6^ | mol (l mito vol)^-1^ mV^-1^ | | b) | (Dash and Beard, 2008) |  |
| Thermodynamic parameters | |  |  | |  |  |  |
| F | Faraday's constant | 0.096 | kJ mol^-1^ mV^-1^ | |  |  |  |
| R | Universal gas constant | 8314x10^-6^ | kJ mol^-1^ K^-1^ | |  |  |  |
| T | Temperature | 310.15 | K | |  |  |  |
|  |  |  |  | |  |  |  |
| Gibbs free energy | |  |  | |  |  |  |
| dG_C10_ | Gibbs free energy for complex I/II reaction at pH 7 | -69.37 | kJ mol^-1^ | | b) | (Dash and Beard, 2008) |  |
| dG_C30_ | Gibbs free energy for complex III reaction | -32.53 | kJ mol^-1^ | | b) | (Dash and Beard, 2008) |  |
| dG_C40_ | Gibbs free energy for complex IV reaction | -122.94 | kJ mol^-1^ | | b) | (Dash and Beard, 2008) |  |
| dG_F10_ | Gibbs free energy for ATP synthase reaction | 36.03 | kJ mol^-1^ | | b) | (Dash and Beard, 2008) |  |
| n_A_ | Number of protons pumped by ATP synthase | 3 | Unitless | | b) | (Dash and Beard, 2008) |  |
|  |  |  |  | |  |  |  |
| Cytosolic ion/nucleotide concentrations and pH | |  |  | |  |  |  |
| pH_e_ | pH (cytosol) | 7.4 | Unitless | | a) | (Bolshakov et al., 2008) |  |
| H_e_ | H^+^ concentration (cytosol) | 10^-pHe^ | M | | b) | (Huber et al., 2011) |  |
| K_ei_ | K^+^ concentration (cytosol) | 120x10^-3^ | M | | a) | (Liu et al., 2003) |  |
| Mg_tot_ | Mg^2+^ concentration (cytosol) | 20x10^-3^ | M | | a) | (Kubota et al., 2005) |  |
| Pi_e_ | Phosphate (Pi) concentration (cytosol) | 20x10^-3^ | M | | b) | (Huber et al., 2011) |  |
| state_fact | Maintains steady-state ATP:ADP at 10:1 | 10/11 | Unitless | | b) | (Huber et al., 2011) |  |
| ATP_e | Steady-state ATP concentration (cytosol) | state_fact*ADTP__tot_ | M | |  |  |  |
| ADP_e_ | Steady-state ADP concentration (cytosol) | ADTP__tot_-ATP__e_ | M | |  |  |  |
| AMP_e_ | AMP concentration (cytosol) | 0 | M | |  |  |  |
|  |  |  |  | |  |  |  |
| Mitochondrial volume/fraction | |  |  | |  |  |  |
| V_mito_ | Mitochondrial Fraction | 0.06 | % | | a) | (Ward et al., 2007) |  |
| W_c_ | Volume fraction: cytosol/mitochondria | 1/V__mito_ |  | |  |  |  |
| W_m_ | Mitochondrial water space | 0.7 | ml water/ml mito | | b) | (Beard, 2005) |  |
| W_x_ | Volume fraction: matrix/mitochondria | 0.9*W_m_ | ml water/ml mito | | b) | (Beard, 2005) |  |
| W_i_ | Volume fraction: IMS/mitochondria | 0.1*W_m_ | ml water/ml mito | | b) | (Beard, 2005) |  |
|  |  |  |  | |  |  |  |
| Potassium uniport and adenylate kinase (neglected) | | |  | |  | (Beard, 2005; Huber et al., 2011) |  |
| x_K_ | Passive potassium transporter activity | 0 |  | |  |  |  |
| x_AK_ | Adenylate kinase activity | 0 |  | |  |  |  |
| K_AK_ |  | 0 |  | |  |  |  |
|  |  |  |  | |  |  |  |
| Moiety conservations | |  |  | |  |  |  |
| NAD_x_ | Total NAD - reduced NADH (matrix) | NAD_tot_ - NADH_x_ |  | |  |  |  |
| Q | Total ubiquinol - reduced ubiquinol | Q_tot_ - QH_2_ |  | |  |  |  |
| C_ox_ | IMS Total cyt *c* - IMS reduced cyt *c* | C_tot_ - C_red_ |  | |  |  |  |
| ATP_fx_ | Free ATP_x_ | ATP_x_ - ATP_mx_ |  | |  |  |  |
| ADP_fx_ | Free ADP_x_ | ADP_x_ - ADP_mx_ |  | |  |  |  |
| ATP_fi_ | Free ATP_i_ | ATP_i_ - ATP_mi_ |  | |  |  |  |
| ADP_fi_ | Free ADP_i_ | ADP_i_ - ADP_mi_ |  | |  |  |  |
| ADP_me_ | Mg-bound ADP |  | | $\frac{\left( K_{DD}+ADP_{e}+Mg_{tot} \right)-\sqrt{\left( K_{DD}+ADP_{e}+Mg_{tot} \right)^{2}-4\left( Mg_{tot}*ADP_{e} \right)}}{2}$ | | |  |
| ADP_fe_ | Free cytosolic ADP | ADP_e_ - ADP_me_ |  | |  |  |  |
| Mg_e_ | Free Mg^2+^ concentration | Mg_tot_ - ADP_me_ |  | |  |  |  |
| Mg_i_ | Mg^2+^ (IMS)^1^ | Mg_e_ |  | |  |  |  |
| K_i_ | K^+^ (IMS)^1^ | K_ei_ |  | |  |  |  |
|  |  |  |  | |  |  |  |
| Parameter for Adenine Nucleotide Transferase (ANT) | |  |  | |  |  |  |
| Psi_x_ |  | -0.65*dPsi |  | | b) | (Korzeniewski and Brown, 1998) |  |
| Psi_i_ |  | 0.35*dPsi |  | | b) | (Korzeniewski and Brown, 1998) |  |

*Suffixes: x, mitochondrial matrix; i, mitochondrial intermembrane space (IMS); c, cytosolic; e, cytosolic (external); m, Mg^2+^ bound; red, reduced; tot, total*

*Abbreviations: MOM, mitochondrial outer membrane; Pi, phosphate*

*^1^* K^+^ and Mg^2+^ ions rapidly equilibrate across OM, so IMS concentration assumed to be equal to cytosolic concentration

^2^ Proton permeability across MOM is assumed >> than permeability of nucleotides and phosphate

*a) Value set ~according to literature*

*b) Value retained from previous publications utilising to model*

*c) Value adjusted during model calibration to in-house measurements*

**Supplementary table 5: Mitochondrial inhibitors and concentrations used in this study.**

| **Drug** | **Mitochondrial target** | **Concentrations** |
| --- | --- | --- |
| **Rotenone** | Inhibits complex I | 2 μM |
| **Antimycin A** | Inhibits complex III | 1 μM |
| **Oligomycin** | Inhibits F_1_F_o_ ATP synthase | 2 μg/ml |
| **FCCP** | Mitochondrial uncoupler | 0.5 μM |

**Supplementary table 6: List of antibodies used in this study**

| **Target protein** (subunit/isoform) | **Experiment** | **Dilution** | **Manufacturer; reference #** |
| --- | --- | --- | --- |
| Actin (AC-40) | WB | 1/1000 | Sigma; A4700 |
| Hsp90 | WB | 1/1000 | BD Biosciences; 610419 |
| Glut3 (B6) | WB | 1/500 | Santa Cruz Biotechnology; sc74497 |
| HK1 | WB | 1/1000 | ThermoFisher Scientific, MA5-14789 |
| LDH (A) | WB | 1/2000 | Novus; NBP1-48336 |
| MPC1 | WB | 1/1000 | Sigma; HPA045119 |
| MPC2 | WB | 1/1000 | Sigma; HPA056091 |
| Complex II, III, ATP synthase | WB | 1/500 | Mitosciences; MS601 (MitoProfile® Total OXPHOS Antibody Cocktail) |
| Complex IV (subunit I) | WB | 1/1000 | Novex; #459600 |
| Complex I (Ndufs1) | WB | 1/2000 | Abcam; ab157221 |
| GFAP | IF | 1/500 | Dako; Z0334 |
| NF200 | IF | 1/200 | Sigma; N5389 |

*WB: Western blot; IF: Immunofluorescence*

**Extended References**

Abramov AY, Scorziello A, Duchen MR (2007). Three distinct mechanisms generate oxygen free radicals in neurons and contribute to cell death during anoxia and reoxygenation. *J Neurosci*. *27*, 1129-1138.

Aguilar-Arnal L, Ranjit S, Stringari C, Orozco-Solis R, Gratton E, Sassone-Corsi P (2016). Spatial dynamics of SIRT1 and the subnuclear distribution of NADH species. *Proc Natl Acad Sci U S A*.

Aubert A, Costalat R (2005). Interaction between astrocytes and neurons studied using a mathematical model of compartmentalized energy metabolism. *J Cereb Blood Flow Metab*. *25*, 1476-1490.

Beard DA (2005). A biophysical model of the mitochondrial respiratory system and oxidative phosphorylation. *PLoS Comput Biol*. *1*, e36.

Bilan DS, Matlashov ME, Gorokhovatsky AY, Schultz C, Enikolopov G, Belousov VV (2014). Genetically encoded fluorescent indicator for imaging NAD(+)/NADH ratio changes in different cellular compartments. *Biochim Biophys Acta*. *1840*, 951-957.

Blacker TS, Mann ZF, Gale JE, Ziegler M, Bain AJ, Szabadkai G, Duchen MR (2014). Separating NADH and NADPH fluorescence in live cells and tissues using FLIM. *Nat Commun*. *5*, 3936.

Bolshakov AP, Mikhailova MM, Szabadkai G, Pinelis VG, Brustovetsky N, Rizzuto R, Khodorov BI (2008). Measurements of mitochondrial pH in cultured cortical neurons clarify contribution of mitochondrial pore to the mechanism of glutamate-induced delayed Ca2+ deregulation. *Cell Calcium*. *43*, 602-614.

Cloutier M, Bolger FB, Lowry JP, Wellstead P (2009). An integrative dynamic model of brain energy metabolism using in vivo neurochemical measurements. *J Comput Neurosci*. *27*, 391-414.

Connolly NMC, Theurey P, Adam-Vizi V, Bazan NG, Bernardi P, Bolanos JP, Culmsee C, Dawson VL, Deshmukh M, Duchen MR, Dussmann H, Fiskum G, Galindo MF, Hardingham GE, Hardwick JM, Jekabsons MB, Jonas EA, Jordan J, Lipton SA, Manfredi G, Mattson MP, McLaughlin B, Methner A, Murphy AN, Murphy MP, Nicholls DG, Polster BM, Pozzan T, Rizzuto R, Satrustegui J, Slack RS, Swanson RA, Swerdlow RH, Will Y, Ying Z, Joselin A, Gioran A, Moreira Pinho C, Watters O, Salvucci M, Llorente-Folch I, Park DS, Bano D, Ankarcrona M, Pizzo P, Prehn JHM (2017). Guidelines on experimental methods to assess mitochondrial dysfunction in cellular models of neurodegenerative diseases. *Cell Death Differ*.

Dash RK, Beard DA (2008). Analysis of cardiac mitochondrial Na+-Ca2+ exchanger kinetics with a biophysical model of mitochondrial Ca2+ handling suggests a 3:1 stoichiometry. *J Physiol*. *586*, 3267-3285.

DiNuzzo M, Mangia S, Maraviglia B, Giove F (2010). Changes in glucose uptake rather than lactate shuttle take center stage in subserving neuroenergetics: evidence from mathematical modeling. *J Cereb Blood Flow Metab*. *30*, 586-602.

Dmitriev RI, Borisov SM, Kondrashina AV, Pakan JM, Anilkumar U, Prehn JH, Zhdanov AV, McDermott KW, Klimant I, Papkovsky DB (2015). Imaging oxygen in neural cell and tissue models by means of anionic cell-permeable phosphorescent nanoparticles. *Cell Mol Life Sci*. *72*, 367-381.

D'Orsi B, Kilbride SM, Chen G, Perez Alvarez S, Bonner HP, Pfeiffer S, . . . Prehn JH (2015). Bax regulates neuronal Ca2+ homeostasis. *J Neurosci*. *35*, 1706-1722.

Gerencser AA, Chinopoulos C, Birket MJ, Jastroch M, Vitelli C, Nicholls DG, Brand MD (2012). Quantitative measurement of mitochondrial membrane potential in cultured cells: calcium-induced de- and hyperpolarization of neuronal mitochondria. *J Physiol*. *590*, 2845-2871.

Hardie DG, Hawley SA (2001). AMP-activated protein kinase: the energy charge hypothesis revisited. *Bioessays*. *23*, 1112-1119.

Heikal AA (2010). Intracellular coenzymes as natural biomarkers for metabolic activities and mitochondrial anomalies. *Biomark Med*. *4*, 241-263.

Huber HJ, Connolly NM, Dussmann H, Prehn JH (2012). A structured approach to the study of metabolic control principles in intact and impaired mitochondria. *Mol Biosyst*. *8*, 828-842.

Huber HJ, Dussmann H, Kilbride SM, Rehm M, Prehn JH (2011). Glucose metabolism determines resistance of cancer cells to bioenergetic crisis after cytochrome-c release. *Mol Syst Biol*. *7*, 470.

Katsura K, Rodriguez de Turco EB, Folbergrova J, Bazan NG, Siesjo BK (1993). Coupling among energy failure, loss of ion homeostasis, and phospholipase A2 and C activation during ischemia. *J Neurochem*. *61*, 1677-1684.

Kipanyula MJ, Contreras L, Zampese E, Lazzari C, Wong AK, Pizzo P, Fasolato C, Pozzan T (2012). Ca2+ dysregulation in neurons from transgenic mice expressing mutant presenilin 2. *Aging Cell*. *11*, 885-893.

Korzeniewski B, Brown GC (1998). Quantification of the relative contribution of parallel pathways to signal transfer: application to cellular energy transduction. *Biophys Chem*. *75*, 73-80.

Kubota T, Shindo Y, Tokuno K, Komatsu H, Ogawa H, Kudo S, Kitamura Y, Suzuki K, Oka K (2005). Mitochondria are intracellular magnesium stores: investigation by simultaneous fluorescent imagings in PC12 cells. *Biochim Biophys Acta*. *1744*, 19-28.

Liu D, Slevin JR, Lu C, Chan SL, Hansson M, Elmer E, Mattson MP (2003). Involvement of mitochondrial K+ release and cellular efflux in ischemic and apoptotic neuronal death. *J Neurochem*. *86*, 966-979.

Murphy MP (2009). How mitochondria produce reactive oxygen species. *Biochem J*. *417*, 1-13.

Nelson DL, Lehninger AL, Cox MM (2008). *Lehninger Principles of Biochemistry*. New York: W. H. Freeman.

Nicholls DG (2006). Simultaneous monitoring of ionophore- and inhibitor-mediated plasma and mitochondrial membrane potential changes in cultured neurons. *J Biol Chem*. *281*, 14864-14874.

Nicholls DG, Budd SL (2000). Mitochondria and neuronal survival. *Physiol Rev*. *80*, 315-360.

Ozmen L, Albientz A, Czech C, Jacobsen H (2009). Expression of transgenic APP mRNA is the key determinant for beta-amyloid deposition in PS2APP transgenic mice. *Neurodegener Dis*. *6*, 29-36.

Richards JG, Higgins GA, Ouagazzal AM, Ozmen L, Kew JN, Bohrmann B, Malherbe P, Brockhaus M, Loetscher H, Czech C, Huber G, Bluethmann H, Jacobsen H, Kemp JA (2003). PS2APP transgenic mice, coexpressing hPS2mut and hAPPswe, show age-related cognitive deficits associated with discrete brain amyloid deposition and inflammation. *J Neurosci*. *23*, 8989-9003.

Schindelin J, Arganda-Carreras I, Frise E, Kaynig V, Longair M, Pietzsch T, Preibisch S, Rueden C, Saalfeld S, Schmid B, Tinevez JY, White DJ, Hartenstein V, Eliceiri K, Tomancak P, Cardona A (2012). Fiji: an open-source platform for biological-image analysis. *Nat Methods*. *9*, 676-682.

Shuttleworth CW (2010). Use of NAD(P)H and flavoprotein autofluorescence transients to probe neuron and astrocyte responses to synaptic activation. *Neurochem Int*. *56*, 379-386.

Sipos I, Tretter L, Adam-Vizi V (2003). Quantitative relationship between inhibition of respiratory complexes and formation of reactive oxygen species in isolated nerve terminals. *J Neurochem*. *84*, 112-118.

Telford JE, Kilbride SM, Davey GP (2009). Complex I is rate-limiting for oxygen consumption in the nerve terminal. *J Biol Chem*. *284*, 9109-9114.

Tottene A, Moretti A, Pietrobon D (1996). Functional diversity of P-Type and R-Type calcium channels in rat cerebellar neurons. *J Neurosci. 16*, 6353-6363.

Ward MW, Huber HJ, Weisova P, Dussmann H, Nicholls DG, Prehn JH (2007). Mitochondrial and plasma membrane potential of cultured cerebellar neurons during glutamate-induced necrosis, apoptosis, and tolerance. *J Neurosci*. *27*, 8238-8249.

Ward MW, Rego AC, Frenguelli BG,… Nicholls DG (2000). Mitochondrial membrane potential and glutamate excitotoxicity in cultured cerebellar granule cells. *J Neurosci*. *20*, 7208-7219.

Wei AC, Aon MA, O’Rourke B, Winslow RL, Cortassa S (2011). Mitochondrial Energetics, pH Regulation, and Ion Dynamics: A Computational Experimental Approach. *Biophys J. 100*, 2894-2903.

Yoshida T, Kakizuka A, Imamura H (2016). BTeam, a Novel BRET-based Biosensor for the Accurate Quantification of ATP Concentration within Living Cells. *Sci Rep*. *6*, 39618.
